# Deciphering the oviductal extracellular vesicles content across the estrous cycle: implications for the gametes-oviduct interactions and the environment of the potential embryo

**DOI:** 10.1186/s12864-018-4982-5

**Published:** 2018-08-22

**Authors:** C. Almiñana, G. Tsikis, V. Labas, R. Uzbekov, J. C. da Silveira, S. Bauersachs, P. Mermillod

**Affiliations:** 1 Department for Farm Animals, University of Zurich, Genetics and Functional Genomics, Clinic of Reproductive Medicine, VetSuisse Faculty Zurich, Zurich, Switzerland; 2 UMR85 PRC, INRA, CNRS 7247, Université de Tours, IFCE, 37380 Nouzilly, France; 3 Plate-forme CIRE, Pôle d’Analyse et d’Imagerie des Biomolécules, INRA, CHRU de Tours, Université de Tours, 37380 Nouzilly, France; 4 Laboratoire Biologie Cellulaire et Microscopie Electronique, Faculté de Médecine, Université François Rabelais, 10 boulevard Tonnellé, 37032 Tours, France; 5 Faculty of Bioengineering and Bioinformatics, Moscow State University, 119992 Moscow, Russia; 6 Department of Veterinary Medicine, University of Sao Paulo, Pirassununga, Sao Paulo Brazil

**Keywords:** Exosomes, Microvesicles, Oviduct, Hormones, Gamete/embryo-maternal interaction, Proteomics, RNA-sequencing

## Abstract

**Background:**

The success of early reproductive events depends on an appropriate communication between gametes/embryos and the oviduct. Extracellular vesicles (EVs) contained in oviductal secretions have been suggested as new players in mediating this crucial cross-talk by transferring their cargo (proteins, mRNA and small ncRNA) from cell to cell. However, little is known about the oviductal EVs (oEVS) composition and their implications in the reproductive success. The aim of the study was to determine the oEVs content at protein, mRNA and small RNA level and to examine whether the oEVs content is under the hormonal influence of the estrous cycle.

**Results:**

We identified the presence of oEVs, exosomes and microvesicles, in the bovine oviductal fluid at different stages of the estrous cycle (postovulatory-stage, early luteal phase, late luteal phase and pre-ovulatory stage) and demonstrated that their composition is under hormonal regulation. RNA-sequencing identified 903 differentially expressed transcripts (FDR < 0.001) in oEVs across the estrous cycle. Moreover, small RNA-Seq identified the presence of different types of ncRNAs (miRNAs, rRNA fragments, tRNA fragments, snRNA, snoRNA, and other ncRNAs), which were partially also under hormonal influence. Major differences were found between post-ovulatory and the rest of the stages analyzed for mRNAs. Interesting miRNAs identified in oEVs and showing differential abundance among stages, miR-34c and miR-449a, have been associated with defective cilia in the oviduct and infertility. Furthermore, functional annotation of the differentially abundant mRNAs identified functions related to exosome/vesicles, cilia expression, embryo development and many transcripts encoding ribosomal proteins. Moreover, the analysis of oEVs protein content also revealed changes across the estrous cycle. Mass spectrometry identified 336 clusters of proteins in oEVs, of which 170 were differentially abundant across the estrous cycle (*p*-value< 0.05, ratio < 0.5 or ratio > 2). Our data revealed proteins related to early embryo development and gamete-oviduct interactions as well as numerous ribosomal proteins.

**Conclusions:**

Our study provides with the first molecular signature of oEVs across the bovine estrous cycle, revealing marked differences between post- and pre-ovulatory stages. Our findings contribute to a better understanding of the potential role of oEVs as modulators of gamete/embryo-maternal interactions and their implications for the reproductive success.

**Electronic supplementary material:**

The online version of this article (10.1186/s12864-018-4982-5) contains supplementary material, which is available to authorized users.

## Background

Exosomes and microvesicles, collectively called extracellular vesicles (EVs) are membrane-enclosed particles loaded with a selection of proteins, lipids, and genetic materials such as DNA, messenger RNA (mRNA), and small non-coding RNA. By transferring this special cargo from one cell to another, EVs play an important role in cell-to-cell communication [1] exerting direct or indirect effects into the recipient cells [2]. EVs have been isolated from most cell types and biological fluids such as urine, saliva, nasal and bronchial lavage fluid, breast milk, plasma and serum [3] and also, in reproductive fluids (seminal, follicular, uterine, oviductal fluids) [4–7]. EVs from the oviductal fluid (oEVS), also known as oviductosomes, have gained considerable attention in the recent years since they might act as natural nanoshuttles, bringing key components from the oviduct into gametes/embryos and could play important roles in sperm capacitation, fertilization and early embryo development [7, 9].

Thus, unraveling the molecular content of the oEVS seems an important requisite to understand the possible roles of the EVs in the gamete/embryo-oviduct dialog and their implications in the reproductive success. To the best of our knowledge, only the oEVs protein content have been examined to date. Two proteins, plasma membrane calcium-transporting ATPase 4 (PMCA4) and Sperm Adhesion Molecule 1 (SPAM1), associated to essential steps before fertilization have been identified in oviductosomes [7, 10]. Besides, our laboratory identified 319 proteins in bovine oEVs collected after ovulation and revealed a group of proteins involved in sperm-oocyte binding, fertilization and embryo development [9]. Furthermore, we demonstrated that the co-incubation of oEVs with in vitro produced embryos increased blastocyst rates, extended embryo survival over time and improved embryo quality [9], proving the functional impact of these vesicles in supporting embryo development. In the light of these results, our efforts in this study have been directed towards a deeper characterization of the oEVs cargo at mRNA, protein, and small RNA level. Since proteins contained in oEVs could have a direct impact on gametes/embryos after the uptake [11] while RNA and small ncRNAs might have an indirect effect by leading to the generation of functional proteins or regulating the sperm/embryo transcriptome, phenotype [12, 13] and their fate.

On the other hand, it is extensively known that steroid hormones induce significant changes in the oviductal transcriptome [14, 15] and in the oviductal fluid composition across the estrous cycle [16, 17]. These changes are essential to provide the optimal environment for sperm storage, capacitation, gamete transport, fertilization and early embryo development [18]. Thus, it is very likely that the oEVs’ composition might also be under regulation of ovarian hormones, however, the impact of these changing regulatory environment on the oEVs secretion and composition is still unknown. Recently, Greening et al.*,* demonstrated that the protein content of uterine EVs released by human endometrial epithelial cells during in vitro culture was altered by estrogen and progesterone treatment, suggesting hormone-specific changes of EVs cargo [19].

Therefore, the objective of the present study was to determine the hormonal regulatory effect of the estrous cycle on the bovine oEVs content at the protein, messenger RNA (mRNA) and small ncRNA level. Deciphering the oviduct EVs content across the estrous cycle will reveal the potential role of these vesicles during the gamete/embryo-oviductal dialogue and their contribution during different stages of the estrous cycle to the early reproductive success. Moreover, the knowledge derived from our study will open up new possibilities for novel, exosome/microvesicles-based treatments of pregnancy failure and infertility.

## Results

### Characterization and quantification of oviduct EVs across the bovine estrous cycle

**Fig. 1:**
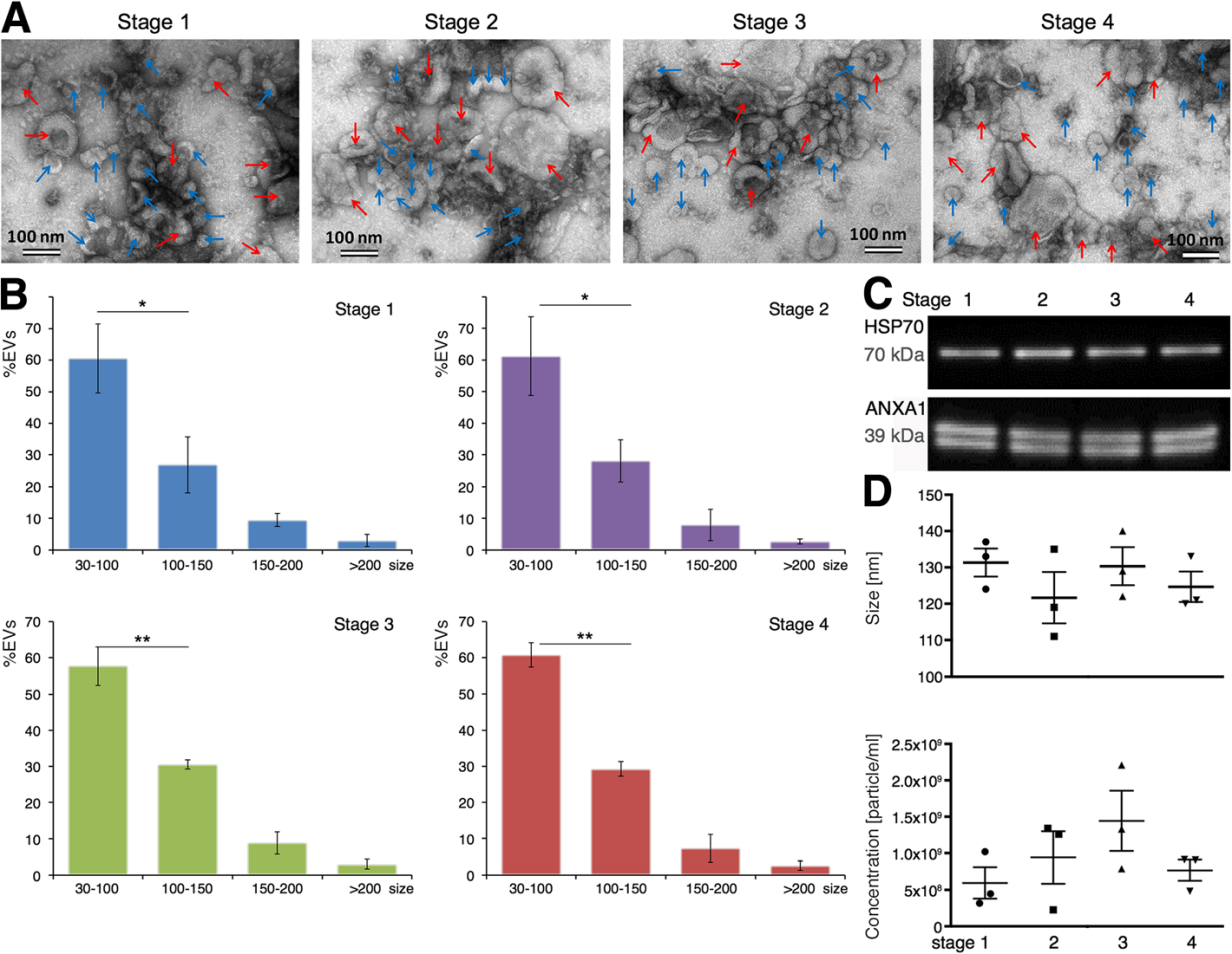
Oviduct EVs characterization and quantification across the bovine estrous cycle. **a** Representative images of exosomes (blue arrows, 30–100 nm size) and microvesicles (red arrows > 100 nm size) across the different stages of the bovine estrous cycle (Stage 1, Stage 2, Stage 3 and Stage 4) by TEM. **b** Histograms showing the size distribution of oEVs across the estrous cycle. A batch of 12 samples containing 3 replicates/stage were analyzed using TEM and ImageJ software. Comparison between populations of exosomes and microvesicles for each stage were performed (*P < 0.05; **P < 0.01, T-test. **c** Western blotting characterization of bovine oEVs at all 4 stages analyzed for known exosomal protein markers. A pool of samples containing 3 biological replicates at the 4 different stages analyzed was used, showing oEVs from all 4 stages were positive for HSP70 and ANAX1.). **d** Bovine oEVs size average and concentration was measured by Nanosight analysis from a different batch of 12 samples (3 replicates /stage)

To obtain a biophysical and molecular characterization of EVs in the oviduct fluid across the estrous cycle, TEM and Western blot analysis were used, respectively. TEM observations confirmed the presence of EVs in bovine oviduct flushings at the 4 stages of the cycle analyzed (Fig. 1a). All preparations from 3 replicates at each stage of the cycle analyzed showed a population of small extracellular vesicles (30-100 nm) resembling exosomes and a population of large extracellular vesicles (> 100 nm) resembling microvesicles. In the literature, microvesicles range from (> 100 up to ∼1000 nm) [20]. Histograms of Fig. 1 (b) show the distribution of exosomes and microvesicles across the estrous cycle. The percentage of exosomes was significantly higher than microvesicles (*p* < 0.05 for Stage 1 and 2, and *p* < 0.01 for Stage 3 and 4) at all stages analyzed.

Western blotting for exosomal protein markers were performed in EVs preparations across the estrous cycle. EVs pool samples from all 4 stages were positive for HSP70, a recognized exosomal protein (positive control) present in 89% of exosome proteomic studies [21, 22] and ANXA1 (Fig. 1c).

In addition, the size and concentration of bovine oviduct EVs was measured by Nanosight nanoparticle tracking analysis from a different batch of 12 samples (3 replicates/stage). Figure 1 (D) shows the size average and concentration for each stage analyzed by Nanosight. No significant differences were observed among stages regarding the size and concentration of EVs.

### Changes in the oviduct EVs molecular content across the bovine estrous cycle

#### Differential mRNA content of oviductal EVs across the estrous cycle

Transcripts derived from a total number of 13,197 genes were identified in the EVs for the analyzed stages of the estrous cycle (after filtering for a minimum number of read counts, see Methods section) (Additional file 1: Table S0). The distribution of the normalized read counts across all transcripts showed that most of the transcripts occurred in low copy numbers and only the top 500 most abundant transcripts/genes showed a frequency of more than 25 transcripts per million. (Additional file 2: Figure S1).

**Fig. 2:**
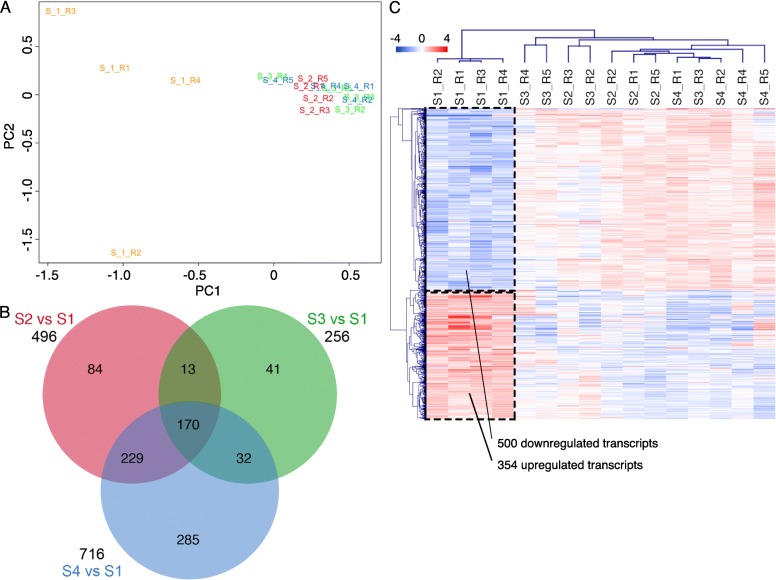
Comparative analysis of the oviduct EVs mRNA content across the bovine estrous cycle. **a** Principal Component analysis (PCA) on mRNA oviduct EVs content at 4 different stages of the estrous cycle (S1 in yellow; S2 in red, S3 in green and S4 in blue). **b** Venn Diagram demonstrating relations among differential transcripts (DT) for selected comparisons during the estrous cycle. Data were analyzed with a cut-off FDR < 0.001. **c** Dendrogram representing results of unsupervised hierarchical clustering (HCL) created with Pearson correlation coefficient by MeV. Rows indicate single differential transcripts (DT), while columns represent individual samples collected at different stages of the estrous cycle. Mean-centered expression values (log2 counts per million of sample – mean of log2 counts per million of all samples) for the samples of the 4 stages are shown. Color scale is from −4 (blue, lower than mean) to 4 (red, higher than mean)

Multidimensional scaling plots (principal component analysis) of normalized read count data revealed a clear separation of the mRNA profiles of stage 1 (S1) EVs to the other stages analyzed (Fig. 2a). Based on this finding, a statistical analysis in comparison of the stages S2, S3, and S4 to S1 was performed. The total number of differential transcripts (FDR 0.001) for these comparisons was 854 (Additional file 1: Table S4). The overlap of differential transcripts between the comparisons is shown in the Venn diagram (Fig. 2b). Hierarchical cluster analysis of the differential transcripts confirmed that the main difference in mRNA abundance was between S1 and the other stages and that transcript frequency between S2, S3, S4 was more or less similar (Fig. 2c; Additional file 1: Tables S1-S3 and Additional file 2: Figure S2.). About 350 transcripts were more abundant in S1 EVs, while 500 showed increased frequencies in EVs from the other stages.

Functional analysis of the top 500 most abundant mRNAs identified in EVs across the bovine estrous cycle was performed using DAVID functional annotation clustering (list of 500 most abundant transcripts in Additional file 1: Table S5). A complete list of the GO terms with a score ≥ 1.3 for the 500 most abundant mRNAs are provided in Additional file 3 (Table S0). The most descriptive categories grouped by similar GO terms are shown in Table 1. The GO terms with highest enrichment scores were associated to protein translation and transport (63), extracellular vesicles (59.4), gene expression (46.5) and cell adhesion (40.5). Other interesting GO terms were related to mitochondrion/oxidation-reduction, apoptosis, response to stress and immune response. A high number of transcripts of the top 500 was associated with GO categories “response to steroid hormones” and “growth factors”. While several transcripts were related to “developmental process in reproduction”.

**Table 1:** DAVID functional annotation clusters for top 500 most abundant mRNA identified in oEVs

Most descriptive categories of DAVID functional annotation clusters groups by similar GO terms	#mRNA^1^	Score^2^
GO Group 1: Protein translation and transport	236	63
protein transport (145, 2.8)^3^, translation (129, 7.3), ribosome (87, 11.4), protein targeting to ER (81, 28.4), nuclear transport (29, 2.2), regulation of protein localization (40, 1.5)		
GO Group 2: Extracellular vesicles	254	59.4
extracellular exosome (253, 3), extracellular vesicle (253, 3), phagocytosis (13, 1.7)		
GO Group 3: Gene expression	314	46.5
RNA binding (203, 4.4), gene expression (244, 1.7), negative regulation of metabolic process (109, 1.6), negative regulation of transcription, DNA-templated (42, 1.4)		
GO Group 4: Cell adhesion	139	40.5
adherens junction (112, 5.3), focal adhesion (77, 6.5), cadherin binding (51, 5.9)		
GO Group 5: Mitochondrion/oxidation-reduction	131	7.4
mitochondrion (86, 1.7), generation of precursor metabolites and energy (37, 3.6), oxidation-reduction process (49, 1.8), ATP metabolic process (30, 4.5), oxidative phosphorylation (23, 4.2)		
GO Group 6: Apoptosis	87	6
programmed cell death (87, 1.7), apoptotic signaling pathway (40, 2.5), apoptotic mitochondrial changes (10, 3.1)		
GO Group 7: ATPase/GTPase	54	6
nucleoside-triphosphatase activity (51, 2.3), ATPase activity (23, 1.9), GTPase activity (21, 3.2), GTP binding (22, 2.0)		
GO Group 8: Response to stress	172	5
cellular response to stress (78, 1.6), response to oxidative stress (28, 2.6), antioxidant activity (11, 4.9), reactive oxygen species metabolic process (16, 2.6), regulation of response to DNA damage stimulus (10, 2.3)		
GO Group 9: RNA processing/transport	42	3.3
RNA splicing (29, 2.6), mRNA processing (25, 1.9), spliceosomal complex (14, 2.6), RNA localization (14, 2.4), nuclear export (10, 2.0)		
GO Group 10: Immune response, proteolysis	112	3.2
immune response (59, 1.4), regulation of proteolysis (40, 2.1), regulation of mRNA stability (23, 4.8), regulation of cytokine production (26, 1.6), platelet activation (13, 2.9), regulation of peptidase activity (23, 2.1)		
GO Group 11: Cell cycle	64	2.9
mitotic cell cycle (45, 1.7), regulation of cell cycle (40, 1.5)		
GO Group 12: Cytoskeleton	71	2.7
cytoskeleton (50, 1.9), microtubule (21, 1.7), cytoskeleton organization (51, 1.6), actin cytoskeleton organization (28, 1.8), actin filament organization (18, 2.0)		
GO Group 13: Signal transduction, phosphorylation	192	2.6
regulation of protein metabolic process (118, 1.7), signal transduction by protein phosphorylation (38, 1.6), intracellular signal transduction (88, 1.2), phosphorylation (75, 1.2), phosphorus metabolic process (105, 1.2)		
GO Group 14: Chromatin	23	2.6
chromatin DNA binding (11, 3.8), protein-DNA complex assembly (15, 2.3), chromatin assembly (9, 2.0)		
GO Group 15: Development	41	2.3
gland development (22, 1.9), reproductive system development (19, 1.6), developmental process involved in reproduction (25, 1.4)		
GO Group 16: Response to hormones, growth factors, nutrients	80	2.2
response to organic cyclic compound (42, 1.7), response to steroid hormone (20, 1.9), response to nutrient levels (19, 1.7), response to growth factor (28, 1.6), response to glucocorticoid (12, 3.1), response to cAMP (8, 3.0)		
GO Group 17: Cell organization	82	1.8
regulation of organelle organization (47, 1.5), regulation of cellular component organization (76, 1.2), cell leading edge (20, 1.9)		
GO Group 18: Others	68	2.6
energy reserve metabolic process (11, 4.3), glycogen metabolic process (10, 4.6), isomerase activity (13, 2.8), muscle contraction (17, 1.9), organic acid metabolic process (37, 1.5)		

Table shows DAVID functional annotation clusters with a score ≥ 1.3. ^1^number of unique mRNA in each GO group;^2^enrichment score (geometric mean of member’s *p*-values of the corresponding annotation cluster in -log_10_ scale) of the annotation cluster; ^3^in brackets: number of genes and fold enrichment for the functional term

**Fig. 3:**
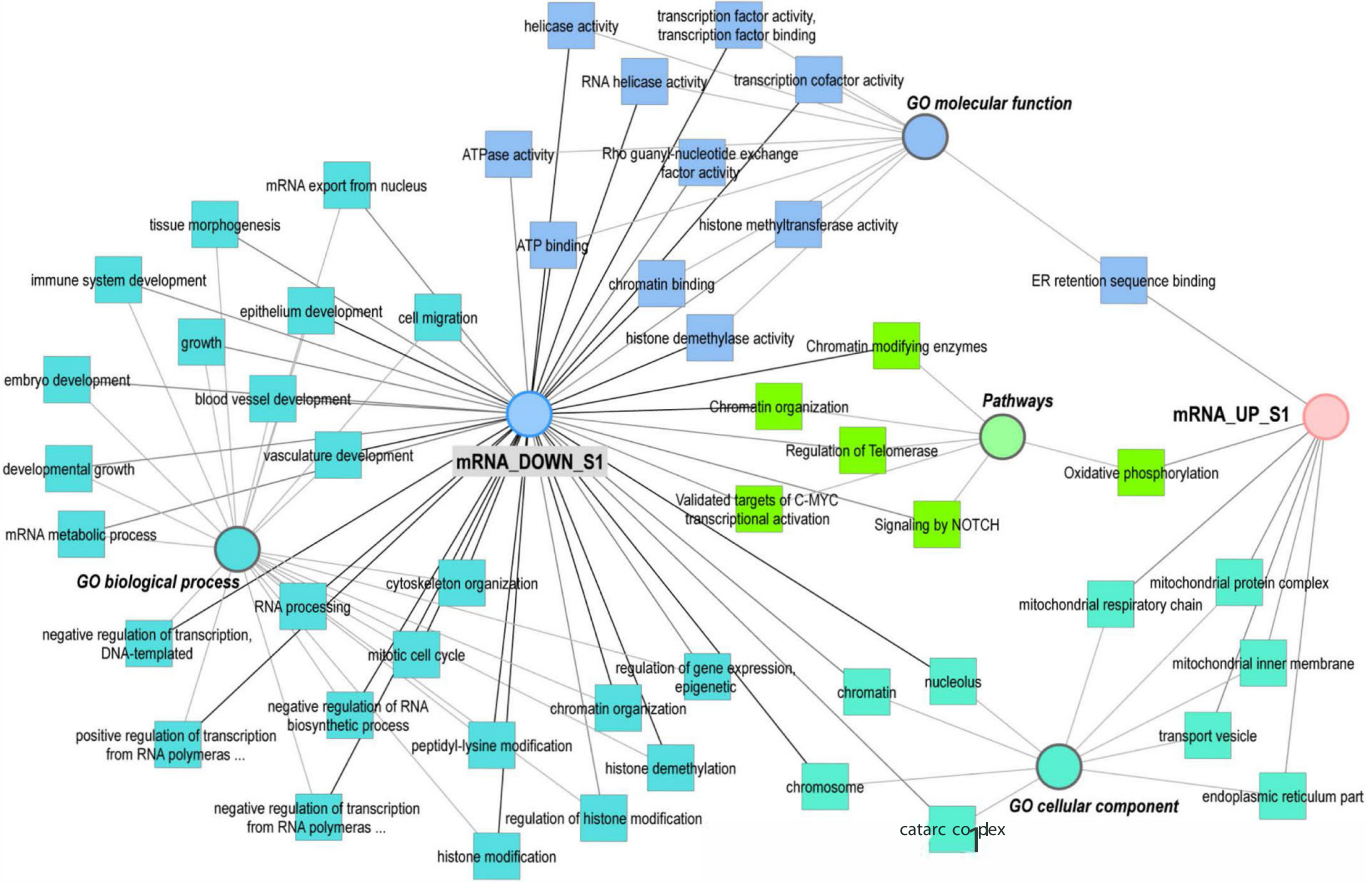
Network of enriched GO terms associated to up and downregulated transcripts during stage 1 (S1). Comparative enrichment analysis of GO terms associated to transcripts up or downregulated in oviduct EVs at stage 1 of the estrous cycle compared to the rest of the stages (S2, S3, S4). The network shows enriched GO terms (biological process, a molecular function and cellular component) and pathways specifically overrepresented for the up and downregulated transcripts

To obtain a more meaningful view of the mRNA EVs content in S1, GO terms of the 500 downregulated transcripts and 354 upregulated transcripts were mapped in a functional annotation network (Fig. 3). A list of the categories shown in the network and assigned genes/transcripts can be found in Additional file 3 (Table S1). Enriched GO terms for upregulated transcripts were related to vesicles and exosomes such as: “extracellular exosome” (number of transcripts: 74), “extracellular vesicle” (74), “membrane-bounded vesicle” (96), “cytoplasmic vesicle” (36), and “membrane vesicles” (17). Upregulated transcripts were also involved in “endoplasmic reticulum” (43), “mitochondrial respiratory chain complex assembly”, and “oxidative phosphorylation” (11)., While downregulated transcripts were significantly enriched in GO terms involved in embryo development such as: “chordate embryonic development”, “embryo development ending in birth” or “egg hatching”, “in utero embryonic development”, “morphogenesis of embryonic epithelium”, and “embryonic epithelial tube formation”. In addition, GO terms such as “cell proliferation”, “cell cycle process”, “cell migration”, “cell motility”, and epithelium and vasculature development were obtained for downregulated transcripts. Moreover, downregulated transcripts were significantly enriched for terms related to regulation of gene expression, epigenetic, regulation of histone modification and demethylation.

#### Differential protein content of oviductal EVs across the estrous cycle

**Fig. 4:**
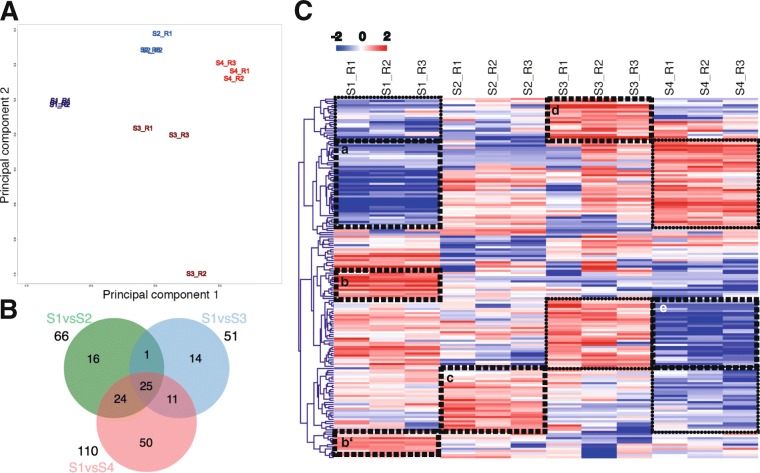
Comparative analysis of the oviduct EVs protein content across the bovine estrous cycle. **A)** Principal Component analysis (PCA) on protein oviduct EVs content at 4 different stages of the estrus cycle (S1 in dark blue; S2 in light blue; S3 in brown and S4 in red) based on normalized weighted spectra data. **B)** Venn Diagram demonstrating relations among differential proteins (DP) for selected comparisons during the estrous cycle. Data were analyzed with cut-off FDR < 0.001. **C)** Dendrogram representing results of unsupervised hierarchical clustering (HCL) by MeV. Mean-centered expression values (log2 expression value of sample – mean of log2 expression values of all samples) for the samples of the 4 stages are shown. Rows indicate single DP, while columns represent different stages of the estrous cycle. Clusters of proteins strongly differentially regulated among stages are pointed with doted squares in HCL figure. These clusters of proteins were used for functional analysis and GO enriched terms are shown in proteins network in Fig. 5

Unlike in our previous study where we analyzed the protein content of the oEVs at post-ovulatory stage [9], here we analyzed the oEVs at different stages of the bovine estrous cycle. Mass spectrometry analysis revealed differential protein abundance of EVs along the bovine estrous cycle. Among the 336 identified clusters of proteins, 170 were differential among different stages of the estrous cycle (*p*-value < 0.05, ratio < 0.5 or > 2). Complete lists of identified and differentially abundant proteins are shown in Additional file 1 (Tables S6-S8). A principal component analysis of normalized weighted spectra data of the top 200 proteins with highest differences across the dataset revealed a clear separation of the protein profiles for the 4 stages (Fig. 4A). Figure 4B represents a Venn Diagram demonstrating the overlaps of differential proteins for selected comparisons during the estrous cycle. A higher number of differentially expressed proteins (DEPs) was found between Stage 4 and Stage 1 (110) than for the rest of the comparisons: S2 vs S1 (66) and S3 and S1 (51). Twenty-five DEPs were overlapping between all comparisons. Hierarchical cluster analysis of the DEPs shows the distinct protein expression profile of EVs across the estrous cycle (Fig. 4C).

Functional annotation of all 336 clusters of proteins identified in oEVs across the bovine estrous cycle was performed using DAVID functional annotation clustering (Additional file 3: Table S2). Most descriptive categories of selected annotation clusters (enrichment score ≥ 1.3) are shown in Table 2. The terms with the highest number of proteins were related to “vesicle”, “exosome”, “secretion”, and “endocytosis”. Other GO terms with high enrichment scores were associated with cell adhesion/junction, protein targeting to ER/protein folding, “regulation of secretion”, and “gene expression”. “Protein biosynthesis”, “posttranscriptional regulation of gene expression”, “regulation of protein modification process” and “protein phosphorylation”, and “regulation of cell communication” were also among the identified overrepresented GO terms. A high number of proteins in EVs were assigned to functional categories related to proteolysis/cell cycle such as: “proteasome complex”, “programmed cell death”, and “apoptosis”. Proteins found in the EVs were also associated to “cell motility/migration”, “response to oxidative stress”, “homeostatic process”, and “epithelial cell differentiation” and “establishment or maintenance of cell polarity”. It is worthy to mention the identification of a set of proteins (21) in EVs related to zona pellucida receptor complex, binding of sperm to zona pellucida and fertilization and steroid binding proteins.

**Table 2:** DAVID functional annotation clusters for all proteins identified in oEVs

Most descriptive categories of DAVID functional annotation clusters groups by similar GO terms	# Proteins^1^	Score^2^
GO Group 1: Vesicle/exosome/secretion	271	114.5
extracellular exosome (263, 4.2)^3^, vesicle membrane (32, 2.8), exocytosis (30, 3.9), secretion (50, 2.4)		
GO Group 2: Cell adhesion/junction	141	49.9
adherens junction (106, 6.9), cell-cell junction (61, 4.3) cell-cell adhesion (71, 3.2), cell junction organization (13, 2.7)		
GO Group 3: Protein secretion/folding	129	29.1
protein targeting to ER (42, 21.1), protein folding (27, 6.2), Protein processing in endoplasmic reticulum (15, 2.8), regulation of secretion (23, 1.8)		
GO Group 4: Gene expression	168	21.1
poly(A) RNA binding (102, 4.3), gene expression (137, 1.4), RNA biosynthetic process (87, 1.2)		
GO Group 5: Wound healing/coagulation	154	9.9
regulation of body fluid levels (38, 4), coagulation (31, 4.5), wound healing (37, 3.6), platelet activation (21, 6.8), blood circulation (19, 5.8)		
GO Group 6: Small molecule binding	109	10.6
nucleoside-triphosphatase activity (47, 3), small molecule binding (96, 1.9), ATP binding (57, 1.9), GTPase activity (22, 4.7)		
GO Group 7: Signal transduction	137	7
posttranscriptional regulation of gene expression (38, 4,2), regulation of protein modification process (62, 2), protein phosphorylation (59, 1.7), regulation of cell communication (85, 1.5)		
GO Group 8: Proteolysis/cell cycle	89	6
proteasome complex (14, 9.6), mitotic cell cycle (37, 3.2), proteolysis (57, 1.7), endopeptidase activity (21, 2.1)		
GO Group 9: Cytoskeleton organization	95	5.4
cytoskeleton organization (45, 2.1), microtubule cytoskeleton (51, 2.1), contractile fiber (15, 3), actin filament organization (19, 3), Ras GTPase binding (13, 2.4)		
GO Group 10: Apoptosis	66	5.2
programmed cell death (61, 1.7), intrinsic apoptotic signaling pathway (12, 2.3), negative regulation of intracellular signal transduction (16, 1.8)		
GO Group 11: Protein stabilization/sperm-zona pellucida interaction	21	4.9
protein stabilization (18, 6.9), zona pellucida receptor complex (6, 30.1), binding of sperm to zona pellucida (7, 10.2), fertilization (10, 3.2)		
GO Group 12: Carbohydrate derivative metabolism	60	3.8
carboxylic acid metabolic process (35, 2.2), NADH metabolic process (9, 12.7), nucleotide metabolic process (29, 2.3), carbohydrate metabolic process (25, 1.7)		
GO Group 13: Protein biosynthesis	19	3.7
Protein biosynthesis (16, 6.7), tRNA aminoacylation (8, 7.9), translational elongation (9, 3.5)		
GO Group 14: Cell motility	52	3.4
locomotion (52, 1.8), cell motility (47, 1.9), cell migration (42, 1.9)		
GO Group 15: Oxidative stress	24	3.4
response to oxidative stress (20, 2.7), reactive oxygen species metabolic process (14, 3.3)		
GO Group 16: Cell development	62	3.1
cell development (51, 1.4), epithelial cell differentiation (20, 1.9), establishment or maintenance of cell polarity (12, 3.7)		
GO Group 17: Ion homeostasis	67	2.2
iron ion homeostasis (6, 4.4), homeostatic process (56, 1.8), ion homeostasis (22, 1.6), regulation of ion transport (21, 2.0)		
GO Group 18: Others	30	7.9
Annexin (9, 40.7), calcium-dependent protein binding (13, 11.2), phospholipid binding (19, 2.7), high-density lipoprotein particle (4, 6.9), steroid binding (6, 3.4)		

Table shows DAVID functional annotation clusters with a score ≥ 1.3. ^1^number of unique proteins in each GO group;^2^highest enrichment score (geometric mean of member’s p-values of the corresponding annotation cluster in -log_10_ scale) of each group; ^3^in brackets: number of genes and fold enrichment for the functional term

**Fig. 5:**
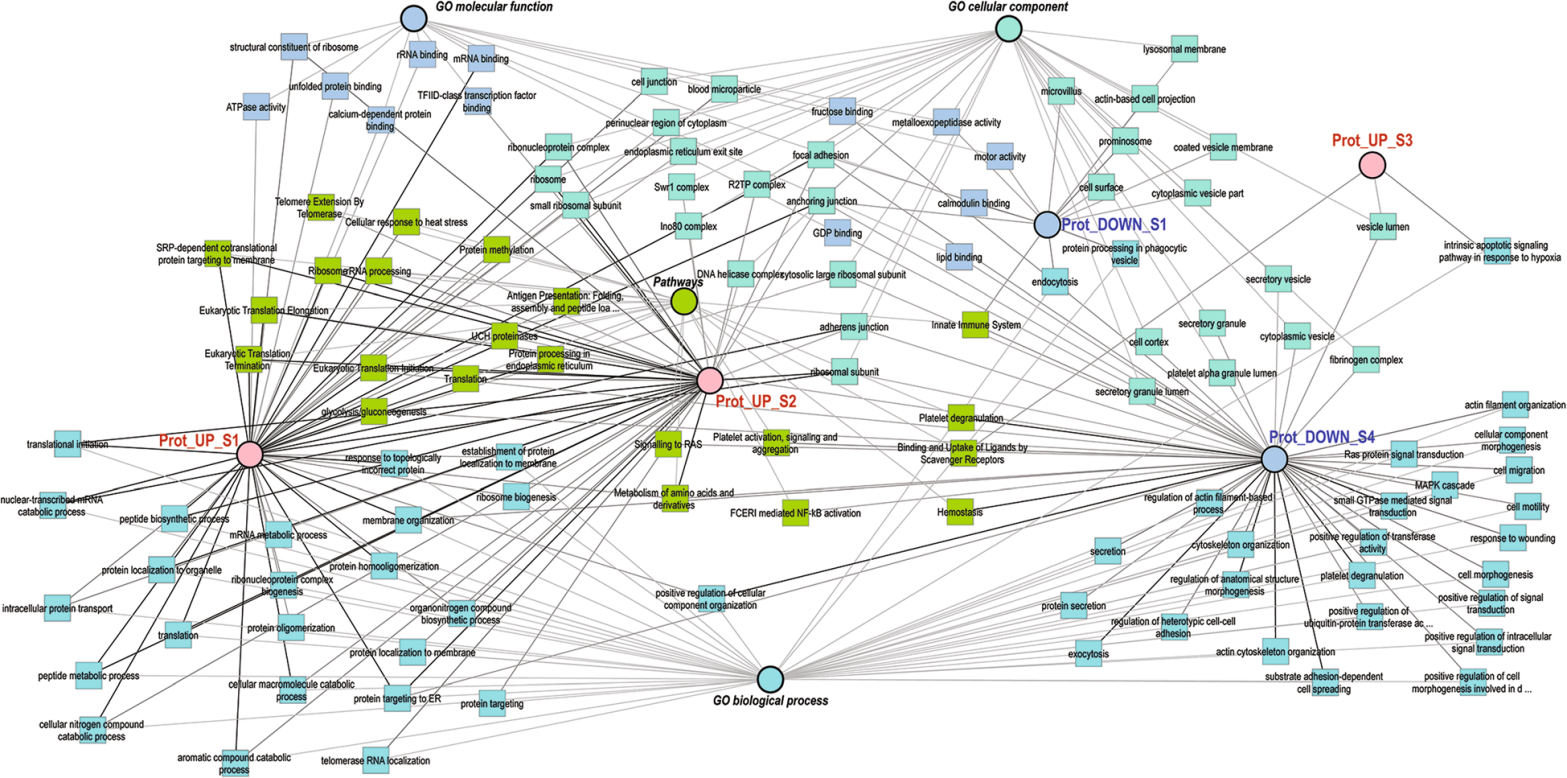
Network of enriched GO terms associated to up and downregulated proteins during stage 1–4. Comparative enrichment analysis of GO terms associated to clusters of differential proteins up or downregulated in oviduct EVs at 1–4 stages of the estrus cycle. The network shows the enriched GO terms (biological process, a molecular function and cellular component) and pathways specifically overrepresented for the different protein cluster lists showing differential expression across the estrous cycle. The 5 clusters of differential proteins used for building the network are represented in Fig. 4C

To illustrate overrepresented functional categories with respect to changes in EVs proteins abundance across the cycle, a network of shared GO terms of the differential proteins is shown in Fig. 5. A list of the categories shown in the network and assigned genes/transcripts can be found in Additional file 3 (Table S3). Five clusters of proteins showing specific profiles across stages were used for this purpose (clusters of proteins are pointed with dotted squares: a, b-b’, c, d and e, in Fig. 4C and listed in Additional file 3: Tables S4-S8). For the cluster of proteins with lower abundance in stage 1 and upregulated in stage 4 (Fig. 4C, a) GO terms related to vesicles, focal adhesion, cytoskeleton, cell surface, metalloexopeptidase activity, and innate immune system were found as overrepresented (Additional file 3: Table S3). The cluster of proteins with highest concentrations during stage 1 (Fig. 4C, b and b’) was enriched for terms representing ribosome biogenesis and protein translation, protein folding, protein transport, protein processing in endoplasmic reticulum, and focal adhesion. Proteins upregulated in stage 2 and downregulated in stage 4 (Fig. 4C, c) were also enriched for functional categories related to ribosome, protein translation and transport, and focal adhesion. Furthermore, proteins of this expression profile were involved in protein targeting, members of DNA helicase complexes, and the proteasome complex. A small set of proteins showed highest abundance in stage 3 (Fig. 4C, d). Specific enrichment was only obtained for vesicle lumen proteins involved in apoptotic signaling and in a scavenger receptor pathway. Furthermore, proteins with lowest abundance in stage 4 and highest in stage 3 were identified (Fig. 4C, e). This group of proteins showed specific enrichment for fructose/lipid/GDP binding, categories related to cytoskeleton organization and cell motility, cytoplasmic vesicles, signal transduction, and secretory processes (Additional file 3: Table S3).

#### Differential small ncRNA content of oviductal EVs across the estrous cycle

The analysis of the small RNA content of oEVs revealed in addition to miRNAs, sequences derived from various classes of non-coding RNAs (ncRNAs). These ncRNAs were ribosomal RNAs (5S, 5.8 S, 18S, 28S, and mitochondrial rRNA), tRNAs, small nucleolar RNAs (snoRNAs, e.g., SNORD, SNORA), small nuclear RNAs (snRNAs, e.g., RN7SK, spliceosomal RNAs), and other ncRNAs (7SL RNA, Y RNA, Vault RNA). Moreover, a very low number of sequences for a few mRNAs was identified (Additional file 1: Table S9). Some of these mRNAs were highly abundant in the mRNA-Seq analysis. The sequences assigned to known or predicted miRNAs comprised 189 different miRNAs based on sequence comparisons to known bovine, human, and porcine miRNAs, miRNA precursors, and predicted precursor sequences (Additional file 1: Table S10).

**Fig. 6:**
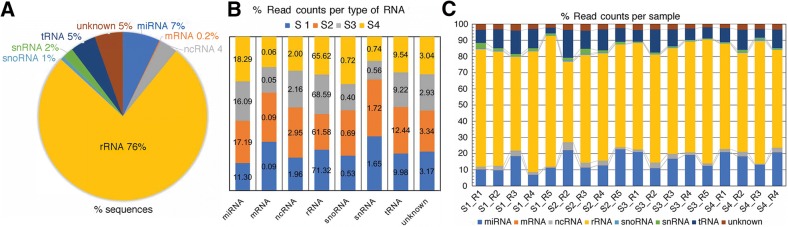
RNA type distribution of the oviduct EVs small ncRNA content across the bovine estrous cycle. **a** Pie graph shows the percentage of sequences attributed to each RNA type among all ncRNAs identified by small RNA sequencing in oviduct EVs across the cycle (miRNAs, rRNA fragments, tRNA fragments, snRNA, snoRNA, and other ncRNAs). **b** Bar graph shows the percent of reads attributed to each RNA type per stage of cycle. **c** Bar graph shows the percent of reads attributed to each RNA type for each sample

RNA type distribution of the small RNA sequencing data content across the estrous cycle showed that the highest percentage of different sequence fragments corresponded to rRNAs (76%) followed by miRNAs (7%, mainly various isoforms of mature miRNAs), unknown sequences, tRNAs, other ncRNAs, snRNAs, snoRNAs, and mRNAs (Fig. 6a). The percentage of read counts attributed to each RNA type per stage of the cycle is shown in Fig. 6b. With respect to read counts, the highest percentage was also found for rRNA fragments (61–71%), miRNAs (11.3–18.3%), and tRNA fragments (9.2–12.4%). The variation between individual samples is represented in Fig. 6c, showing the percentage of reads attributed to each RNA type for each sample.

**Fig. 7:**
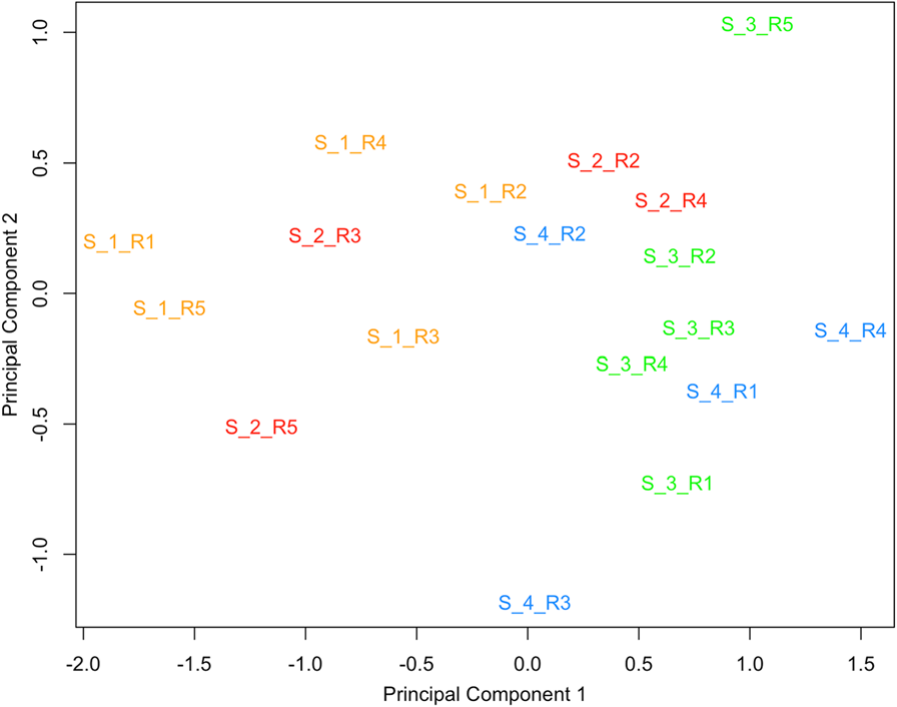
Comparative analysis of the snRNA expression across the bovine estrous cycle. Principal Component analysis (PCA) on snRNA oviduct EVs content at 4 different stages of the estrous cycle (Stage 1: S1 in yellow; Stage 2: S2 in red, Stage 3: S3 in green and Stage 4: S4 in blue) for all snRNA identified. Five different replicates for each stage were analysed (represented by R1, R2, R3, R4 and R5) in the plot. PCA showed a partial separation between stage 1 and 2 (S1, S2) and stages 3 and 4 (S3, S4)

Comparative analysis of all small ncRNA content including rRNA and tRNA fragments and for each individual RNA type across the bovine estrous cycle is represented by several MDS plots in Additional file 2: (Figure S3). In contrast to mRNA and protein EVs content, small ncRNA content did not shown a clear separation for the cycle stages based on all small ncRNA types or the individual RNA types analyzed (rRNA, tRNA, snoRNA, miRNA and other ncRNA). Only for snRNA (Fig. 7), a partial grouping of stage 1 and 2 (S1, S2) and stage 3 and 4 (S3, S4) samples was observed.

When all small ncRNA sequences were analyzed, higher differences were found between stage 4 and stage 1 than between stage 3 and stage 1, with 1036 and 535 differentially abundant sequences (FDR < 0.05), respectively (Additional file 2: Figure S4). While no significant differences were observed for stage 2 compared to stage 1 for any of the individual ncRNA types analyzed. For rRNA fragments, 580 differential sequences were found between stage 4 and stage 1 and 318 between stage 3 and stage 1 FDR (< 0.05) (Additional file 2: Figure S5). For miRNA, Table 3 shows differential miRNAs among stages with 8 differentially regulated miRNAs (20 sequences) between S4 vs S1, while 5 miRNAs (8 sequences) between S3 vs S1 (FDR < 0.05; Additional file 2: Figure S6) (13 differentially regulated miRNAs (39 sequences) S4 vs S1; 9 miRNAs (21 sequences) S3 vs S1; FDR < 1). Only for differential tRNA (Additional file 2: Figure S7) and snRNA (Additional file 2: Figure S8), higher differences were observed between stage 3 and stage 1 than for the comparison of stage 4 and stage 1 (tRNA: 29 (S3 vs S1) and 6 (S4 vs S1); snRNA: 93 (S3 vs S1) and 85 (S4 vs S1), FDR (< 0.05)). Hierarchical cluster analyses of the differential sequences identified for each type of small ncRNA between stage 3–4 and stage 1 are provided in Additional file 2: Figures S4-S9. However, the biological function of differentially abundant rRNA and tRNA fragments is questionable.

**Table 3:** List of differential miRNAs among S3 vs S1 and S4 vs S1 (FDR < 0.1)

Stage comparison	Regulation	
	UP	DOWN
S3 vs S1	**bta-miR-10b**	**bta-miR-449a**
	**bta-miR-125b-2-5p**	**bta-miR-200b-3p**
	bta-miR-92a	**hsa-miR-203a-3p**
	bta-miR-423-3p	bta-miR-375
		bta-miR-429
S4 vs S1	**bta-miR-10b**	**bta-miR-200b-3p**
	**bta-miR-151-3p**	**bta-miR-449a**
	**bta-miR-24-3p**	Novel miRNA
	**bta-miR-28-5p**	bta-miR-34b-3p
	**bta-miR-29c**	
	**bta-miR-423-3p**	
	bta-miR-30d	
	bta-miR-148a-3p	
	bta-miR-125b-2-5p	

All miRNA FDR < 0.1; miRNA FDR < 0.05 in bold

**Fig. 8:**
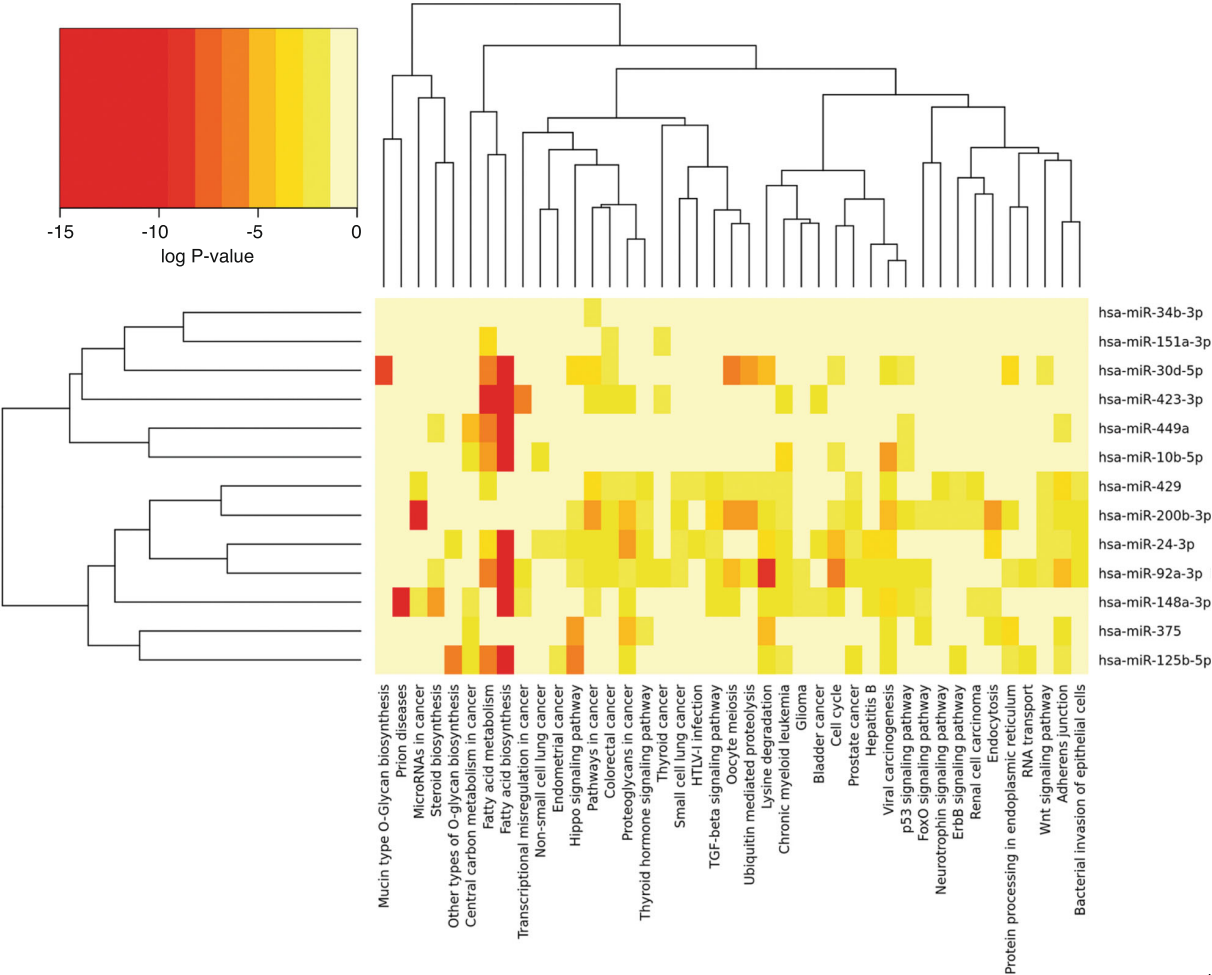
Selected miRNA and their associated pathways. Selected miRNAs based on differential expression and high abundance in EVs in Stage 1 and Stage 4 were used for clustering analysis based on the presence of interactions of target genes and pathways using DIANA-miRPath v3.0. Heatmap clustering of 13 miRNAs versus their associated pathways based on significance levels. Color scale from red (highest significance) to yellow (not significant)

In order to shed some light on the potential impact of EVs’ miRNAs on spermatozoa or early embryos, functional and gene target analyses were performed on 13 selected miRNAs showing differential expression (FDR 0.1) among stages and high abundance in EVs during Stage 1 and Stage 4 (hsa-mir-10b-5p, hsa-miR-423-3p; hsa-miR-449, hsa-miR-375, hsa-miR-24-3p, hsa-miR-429, hsa-miR-148a-3p, hsa-miR-34b-3p, hsa-miR-200b-3p, hsa-miR-92a-3p, hsa-miR-151a-3p, hsa-miR-30d-5p, hsa-miR-125b-5p). For this purpose, human orthologous miRNAs were used for analysis in the DIANA-TarBase v7 tool. The selected miRNAs, pathways involved and the number of genes and gene symbols of the target genes in each pathway are shown in Table 4 and Additional file 3 (Table S9–10). This data was used for cluster analysis based on presence of interactions of target genes and pathways by DIANA-miRPath v3.0. The depicted heatmap shows the 13 selected miRNAs versus pathways based on significance levels (Fig. 8). Interesting pathways targeted by these 13 miRNAs were fatty acid biosynthesis and metabolism; mucin type O-Glycan biosynthesis, cell cycle, focal adhesion, adherens junction, gap junction, thyroid hormone signaling pathway, HIF-1 signaling pathway, p53 signaling pathway, FoxO signaling pathway, TGF-beta signaling pathway, Ras signaling pathway, Wnt signaling pathway, and protein processing in endoplasmic reticulum. Pathways related to reproductive functions were also identified: steroid biosynthesis, progesterone-mediated oocyte maturation and oocyte meiosis.

**Table 4:** Selected miRNAs in oEVs in Stage 1 and 4 and associated pathways and target genes

miRNAs	KEGG pathway	p-value	Examples Target Genes (Gene Symbol)
hsa-mir-10b-5p	Pyrimidine metabolism	0.0214	NME4,NME2,POLR2D,POLR2A,POLR1B,NME1,CMPK1
hsa-miR-423-3p	Fatty acid biosynthesis	0.0000	FASN, ACACA
	Fatty acid metabolism	0.0000	FASN, ACACA, ACOX3, FADS2
hsa-miR-449a	Carbon metabolism	0.0286	TKT,GAPDH,GOT1,H6PD,MDH2,RPIA,ENO1,ALDOA,ADH5
	HIF-1 signaling pathway	0.0390	NFKB1,GAPDH,LTBR,BCL2,ENO1,LDHA,PIK3CA,SLC2A1,MKNK2,ALDOA,SERPINE1
hsa-miR-375	Hippo signaling pathway	0.0000	YAP1,YWHAG,CCND2,MOB1B,YWHAB,WWC1,AMOT,FZD8,MPP5,FZD4,DLG4,
	Amino sugar and nucleotide sugar metabolism	0.0390	PGM2,CMAS,UXS1,UGP2,HK2
hsa-miR-24-3p	Fatty acid biosynthesis	0.0000	FASN,MCAT,ACACA
	Vitamin B6 metabolism	0.0001	PDXK,PNPO,PHOSPHO2
	Endocytosis	0.0005	RNF41,PSD4,CHMP7,VPS45,SMAD2,CBL,DNM2,FGFR3,VTA1,AGAP3,CAV1,AP2B1,N
	Hippo signaling pathway	0.0014	ACTB,GSK3B,FZD5,YWHAH,SMAD2,BTRC,APC,NF2,YWHAG,SMAD3,WWTR1,TGFB1,
	Bacterial invasion of epithelial cells	0.0078	ACTB,ITGB1,CBL,DNM2,PIK3CB,CAV1,PXN,BCAR1,CLTC,CD2AP,CAV2,CTTN,PTK2,
	TGF-beta signaling pathway	0.0228	SMAD2,ACVR1B,SMAD3,TGFB1,SKP1,MYC,EP300,IFNG,E2F4,CREBBP,TGFBR2,BMP4,
	Glycosaminoglycan biosynthesis - heparan sulfate / heparin	0.0343	HS6ST2,NDST1,HS6ST1,EXTL3,HS2ST1,HS6ST3
	DNA replication	0.0343	CNA,FEN1,MCM4,MCM5,MCM7,RNASEH1,PRIM1,POLA1
	RNA degradation	0.0423	PDCP1A,CNOT6,TOB2,CNOT4,DDX6,HSPD1,EXOSC3,EXOSC1,BTG2,DCP2,RQCD1,
hsa-miR-429	Axon guidance	0.0009	PAK2,PPP3R1,RHOA,KRAS,PPP3CA,PTK2,CFL2,RAC1,LIMK1,EFNA1
	Fc gamma R-mediated phagocytosis	0.0350	CRKL,CRK,WASF3,PLCG1,CFL2,RAC1,LIMK1,ARF6
	Steroid biosynthesis	0.0000	SC5D,MSMO1,DHCR24,LSS,FDFT1,LIPA
	Antigen processing and presentation	0.0019	B2M,IFI30,HSPA4,HSP90AA1,HLA-C,HLA-B,NFYA,PSME2,CANX,PDIA3,HSPA8,
	Progesterone-mediated oocyte maturation	0.0039	ADCY1,CCNB1,PGR,ADCY7,CCNA2,CDC25B,CPEB4,HSP90AA1,PDE3A,RAF1,
	Gap junction	0.0346	ADCY1,ADCY7,GNAS,MAP2K5,RAF1,TUBB,TUBB6,CDK1,KRAS,TUBA8,GNA11
hsa-miR-34b-3p	Glycosaminoglycan degradation	0.0000	GNS,IDS,GLB1
hsa-miR-200b-3p	Ras signaling pathway	0.0043	KSR1,RASA2,SHC1,PAK2,ETS1,RALBP1,PLD1,RHOA,KRAS,PLCG1,REL,FLT1,
	Neurotrophin signaling pathway	0.0273	CRKL,CRK,SHC1,FRS2,BCL2,RHOA,KRAS,RPS6KA5,PLCG1,JUN,KIDINS220
hsa-miR-92a-3p	Cell cycle	0.0000	GSK3B,PCNA,SMC1A,CCNB1,SMAD2,HDAC1,CCND2,DBF4,SMC3,CDKN1B,
	Adherens junction	0.0000	ACTB,TGFBR1,WASL,SMAD2,IQGAP1,IGF1R,VCL,FYN,MLLT4,NLK,CDH1,S
	Thyroid hormone signaling pathway	0.0011	ACTB,GSK3B,PRKCA,MED13L,NRAS,RCAN1,PIK3CB,HDAC1,MED13,NOTCH2
	FoxO signaling pathway	0.0024	IRS2,BRAF,STAT3,TGFBR1,CCNB1,SMAD2,NRAS,STK4,GABARAPL2,PIK3CB,SETD7
	RNA transport	0.0025	SAP18,GEMIN6,NUP153,TGS1,NUP210,XPOT,EIF3B,ELAC2,RPP14,EIF2S3,RNPS1
	p53 signaling pathway	0.0045	CCNB1,BID,THBS1,CCND2,CDK1,GADD45A,CDK6,CHEK1,ATM,CCND1,CCNE2,SHISA5
	Signaling pathways regulating pluripotency of stem cells	0.0178	PCGF6,JARID2,TCF3,GSK3B,STAT3,KAT6A,SMAD2,NRAS,INHBB,PIK3CB,WNT5A,TBX3
	Protein processing in endoplasmic reticulum	0.0179	HSPA1A,EIF2AK1,RAD23B,ERO1L,NPLOC4,SEL1L,UBQLN1,SEC23A,SSR2,P4HB,XBP1
	Notch signaling pathway	0.0190	HDAC1,CTBP1,DTX2,NOTCH2,HES1,HDAC2,KAT2A,RBPJ,KAT2B,EP300,CREBBP,MAML1
	Spliceosome	0.0210	HSPA1A,LSM7,DDX23,SRSF5,PPIL1,U2SURP,EFTUD2,SF3B1,U2AF2,SNRPE,SF3A3
	MAPK signaling pathway	0.0210	BRAF,DUSP4,PRKCA,HSPA1A,TGFBR1,NFKB1,NRAS,CRKL,STK4,ELK4,CRK,DUSP6
	Wnt signaling pathway	0.0386	GSK3B,PRKCA,LRP6,TBL1X,BTRC,VANGL1,WNT5A,CTBP1,CCND2,FZD6,AXIN1,SKP1
	Focal adhesion	0.0393	BRAF,ACTB,GSK3B,PRKCA,ITGB8,CRKL,CRK,THBS1,ITGA8,PIK3CB,PIP5K1C,PPP1CC
	Biosynthesis of amino acids	0.0473	TKT,BCAT2,GAPDH,PHGDH,TALDO1,PKM,SHMT2,BCAT1,MTR,ENO1,GOT2,PFKM,ASS1
hsa-miR-151a-3p	Biosynthesis of unsaturated fatty acids	0.0000	PTPLB,ELOVL5,SCD
	Fatty acid metabolism	0.0003	HADH, PTPLB,ACACA,SCD,ELOVL5
hsa-miR-30d-5p	Mucin type O-Glycan biosynthesis	0.0000	GALNT7,B4GALT5,ST3GAL1,GALNT6,GCNT3,GALNT1,GALNT3,GALNT2
	Oocyte meiosis	0.0000	FBXO5,CAMK2D,PGR,BTRC,CALM1,CPEB4,YWHAG,PPP1CC,PPP3R1
	Ubiquitin mediated proteolysis	0.0000	UBE2R2,BTRC,FBXW7,TRIM37,CUL2,NEDD4L,FZR1,PRPF19,MAP3K1
	mRNA surveillance pathway	0.0317	MSI2,SAP18,PPP2R3A,EIF4A3,HBS1L,PPP1CC,CPSF2,CPSF6,PAPOLA
hsa-miR-125b-5p	ErbB signaling pathway	0.0016	PRKCA,ERBB2,NRAS,CRKL,CRK,PAK2,RAF1,CDKN1B,EIF4EBP1,ERBB3
	Regulation of actin cytoskeleton	0.0135	SSH2,EZR,NRAS,CRKL,CRK,PIP5K1C,PAK2,ARHGEF12,PPP1CC,ACTG1

Table shows selected miRNAs based on differential expression and high abundance in EVs in Stage 1 and Stage 4 and associated representative pathways and target genes

### Validation of interesting proteins and miRNA identified in oviductal EVs by western blotting and qPCR

**Fig. 9:**
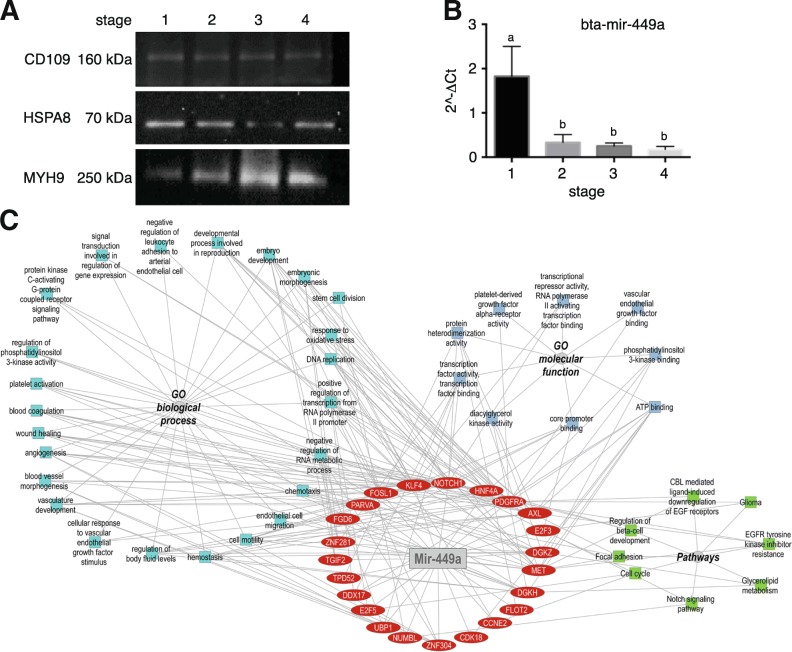
Validation of Mass Spectrometry and miRNA analyses of bovine oEVs by Western immunoblotting and qPCR. **a** Western blot analysis confirmed MS results for 3 proteins associated with reproductive roles and showing differential abundance or not across the bovine estrus cycle: CD109, HSPA8 and MYH9. A pool of samples containing 3 biological replicates at the 4 different stages analyzed was used. **b** qPCR analysis confirmed miRNA-seq results for Mir-449a, which dysregulation has been associated with defective cilia in the oviduct and infertility. Mir-449a was more abundant in S1 compared to the rest of the stages (*p* < 0.05, T-test). A new batch of oEVs isolated from oviducts classified in the four stages of the estrous cycle (*n* = 12) was used. **c** Network of Mir-449a target genes and overrepresented functional categories

Proteomic results were confirmed by Western blot analysis for three of the top 25 proteins most abundant in proteins in oEVs and associated to reproductive functions (CD109, HSPA8 and myosin 9; Fig. 9a). CD109 was not found as differentially regulated across the estrous cycle. HSPA8 was more abundant at S1 compared to the rest of the stages (S2, S3 and S4). By contrast MYH9 was less abundant in S1 than in the stages S2, S3 and S4. These results confirmed the different proteomic profile of oEVs at different stages of the bovine estrous cycle.

The differential regulation of mir-449a in oEVs across estrous cycle detected by RNA-seq analysis was also confirmed by qPCR in a different set of samples (Fig. 9b). Dysregulation of mir-449a has been associated with defective cilia in the oviduct and infertility. Mir-449a was more abundant in S1 compared to the rest of the stages, which may downregulate a set of target genes in S1 in contrast to the other stages of the cycle. To investigate the possible target genes for mir-449a, DIANA tool was used with two different databases (TarBase and TargetScan). TarBase showed 512 target genes for mir-449a, while TargetScan showed 283 genes. Comparative analysis of these two lists of target genes for mir-449a provided 32 common target genes that were used for further functional analysis using ToppCluster tool. Network in Fig. 9c shows the target genes (in red) for mir-449a and the network for GO enriched terms for those target genes. Furthermore, GO terms associated to embryo development, embryonic morphogenesis and stem cell division were identified. Furthermore, target genes were involved in, wound healing, angiogenesis and vasculature development. Besides, genes related to homeostasis, response to oxidative stress and chemotaxis or cell motility were also found. In addition, target genes were involved in cell cycle, focal adhesion and Notch signaling pathways among other.

## Discussion

This study demonstrates that oviductal epithelial cells can release EVs (exosomes and microvesicles) to the oviductal environment at different stages of the estrous cycle and that their molecular cargo is markedly regulated by steroid hormones. So far, only a few studies have investigated the effects of oEVs on murine sperm functionality and early bovine embryo development in vitro [7, 9, 23, 24]. While a systematic analysis of the oEVs content at mRNA, small ncRNA and protein levels to help to understand the effects of oEVs on spermatozoa and embryo was lacking. We did a first approach in our lab by comparing the protein content of in vivo oEVs obtained at the post-ovulatory stage of the bovine estrous cycle and in vitro oEVS obtained from in vitro culture of BOEC and showing a differential protein profile [9]. This study led us to hypothesize that the oEVs content may be under hormonal regulation and suggested to further investigate the oEVs cargo at different levels. Here, we provide the first extensive analysis of the molecular signature of oEVs at transcriptomic and proteomic levels across the bovine estrous cycle.

Recently, Burns et al., [25] showed that the amount of EVs in the ovine uterine lumen increased from day 10 to 14 of estrus cycle and also, after P4 treatment for 14 days, suggesting that P4 may regulate endometrial epithelial production of EVs and their release into the uterine lumen. To evaluate the oEVs concentration along the estrus cycle in our study, two different methods (TEM and Nanosight) with two different batches of samples were used. However, the amount and size distribution of EVs in the oviductal fluid did not vary among the different stages of the estrous cycle (Fig. 1), despite the molecular cargo was under strong hormonal regulation. Differences in species (bovine versus ovine), source of EVs (oviduct versus uterus), window of the cycle (short period 10–14 post-estrus versus 4 stages of the cycle), hormonal induction (natural versus artificial by injecting intramuscularly same amount of P4 during 14 days) could explain the different findings among studies.

### Protein cargo in oviductal EVs is under hormonal regulation

Proteomic analysis showed differential protein profiles across the different stages of the estrous cycle, demonstrating a strong effect of the steroid hormones on oEVs protein cargo. Of the 336 proteins identified in oEVs, 170 were differentially abundant across the estrous cycle and could be grouped in different clusters of proteins based on similar profiles over the cycle stages. Our data are in line with recent studies showing a hormonal effect on the protein cargo of exosomes/EVs derived from the uterus. Greening et al.*,* [19] showed that the exosome protein cargo derived from a human endometrial cell line was also under hormonal regulation. In this case, the authors treated endometrial cells with estrogen (E) or estrogen plus progesterone (E + P) during culture to mimic the hormonal profile in the proliferative and secretory phases of the menstrual cycle. They found 254 proteins as enriched in exosomes produced by E-treated cells while 126 proteins were enriched in exosomes from E + P-treated cells. Moreover, Burns et al., [26] showed a differential protein content in uterine EVs collected from day 14 cyclic sheep (40 unique proteins) compared to pregnant sheep (70 unique proteins), demonstrating also a strong hormonal regulation of the uterine EVs protein content. We compared our list of oEVs proteins with those studies and found that 171 proteins were in common to Greening et al., [19], while only 16 were shared with Burns et al.*,* [26]. It is worth pointing out that our study differs from Greening et al. [19] and Burns [25] in two important aspects: 1) the source of EVs, in our study is the oviduct while the uterus in those studies, differences may point to a specific oviductal EVs signature and 2) we showed a more biological and natural effect, since our samples were collected from cyclic animals and not induced artificially by hormonal treatment in vivo or in vitro.

Among the oEVs proteins identified, we found an important number of proteins in common to other studies [19], which include proteins involved in exosome biogenesis (ESCRT-associated: VPS13C, FAM129B, PDCD6IP) and intracellular vesicle trafficking and release (DYNC1LI1; SNAP23, STXBP1). In addition, tetraspanins (CD9) and GTPases (RAB1A, RAB2A, RAB7A, RAB11A, RAB5C) were identified in oEVs. Several proteins involved in exosome sorting (COPA, CLTC, ARF1, RAC1) were also found. Furthermore, oEVs were enriched in Annexins, another class of proteins commonly seen in exosomes, involved in membrane trafficking and fusion events (ANXA1–8, ANXA10). Oviductal EVs were also enriched for heat-shock proteins (HSP90, HSC70) involved in homeostasis and crucial functions in the biosynthesis, folding/unfolding, transport and folding of proteins [27]. Despite we found a number of proteins characteristic of the exosomal proteomic signature in different cells types/tissues, we also identified proteins characteristic of the oviduct with important roles in the gamete/embryo–oviduct interactions, such OVGP1, together with ANXA2, HSPA8, HSP90, HSP70, Gelsolin and Ezrin, pointing out to an oviductal EVs signature. Particularly OVGP1, it has been identified as one of the major expressed proteins in this organ.

Further comparison of our data with the protein cargo of oviductosomes was difficult, since so far, no available studies have provided an extensive analysis of the oviductosome content in addition to our own studies. The available studies have focused only on the profiles of specific proteins such as: plasma membrane Ca^+ 2^-ATPase 4 (PMCA4) [7] and sperm adhesion molecule 1 (SPAM1) [10]. PMCA4 is involved in regulating progressive and hyperactivated sperm motility, while SPAM1 seems to enhance hyaluronic acid-binding ability and cumulus penetration efficiency, both essential steps for fertilization [7, 10, 28, 29]. It has been shown that PMCA4 is also under the control of female sex hormones, showing elevated levels of the protein in oviductal and uterine fluids in the mouse during proestrus/estrus compared to metestrus/diestrus [7]. However, neither PMCA4 nor SPAM1 were identified in our study at protein or mRNA level. This most likely relates to differences in the methodology to isolate oEVs, the proteomic approach or species-specific differences [30]. However, when we compared the proteins identified here with our previous study [9], where oEVs were only from post-ovulatory stage, we found that 69% of proteins (184 proteins) were in common to both studies.

Furthermore, more differences in oEVs protein cargo were identified between post-ovulatory (S1) and pre-ovulatory (S4) stages (S4 vs S1: 110 proteins) compared to luteal phase (S2 vs S1: 66 proteins and S3 vs S1, 51 proteins). For example, MIF, AQP5, HSPA8, associated to reproductive events [31, 32] were more abundant in EVs of S1 than S4. Our results are in line with other studies showing that the expression of these proteins in oviductal epithelial cells and/or fluid was higher during the post-ovulatory stage when compared to the pre-ovulatory stage [16, 33, 34]. In contrast, MYH9 (myosin heavy chain 9) and CLTC (clathrin heavy chain) proteins were more abundant in EVs during the pre-ovulatory stage in our study. Similar results were found also in the oviductal fluid in cattle and sheep [16, 35]. The proteins identified with higher abundance during the pre-ovulatory period, could play an important role in maintaining sperm viability in the “sperm reservoir” [36]. While the proteins with higher abundance during the post-ovulatory period could be involved in: final maturation of the oocyte and transport to the fertilization place; release of spermatozoids from the sperm reservoir, sperm hyperactivation and sperm transport toward the oocyte; interaction sperm-oocyte and fertilization; and finally, the early development of the embryo [37].

**Fig. 10:**
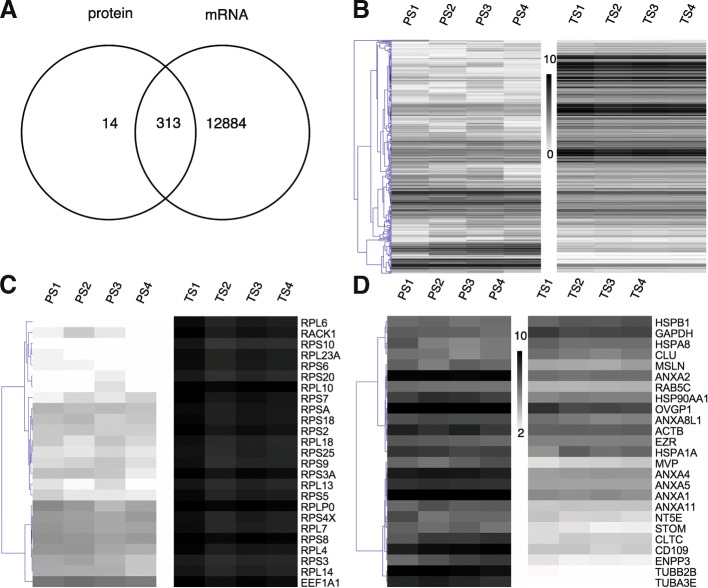
Overlap of all transcripts and proteins identified in oviduct EVs across the bovine estrus cycle. **a** Venn Diagram demonstrating the overlap among all transcripts and proteins identified in EVs; **b** Heatmap representing results of unsupervised hierarchical clustering (HCL) by MeV. Rows indicate single proteins and transcripts, while columns represent different stages of the estrous cycle for transcripts (TS1-TS4) and proteins (PS1-PS4) analyses. Expression values for proteins and transcripts were log2-transformed and scaled to have values in a similar numerical range. **c** Heatmap representing the 25 top most abundant proteins which were attributed to known exosomal proteins (Annexins, Heat Shock proteins) and proteins associated to reproductive roles (OVGP, HSPA8). **d** Heatmap showing the 25 top most abundant transcripts representing mainly mRNAs encoding different ribosomal proteins (RPS, RPL)

Indeed, among the 25 most abundant proteins in oEVs (Fig. 10c), which could exert a major effect when they are taken up by gametes/embryos, we identified 4 heat shock proteins (HSPs): HSPB1, HSPA8, HSP90AA1, HSPA1A. HSPs are among the first proteins produced during embryonic development and are crucial in fertilization and in early embryonic development [38]. Small heat shock protein 25 (HSPB1) plays an important role by acting synergistically with other HSPs during stress conditions to exert cryoprotection and anti-apoptotic effects [39]. Other HSPs identified here such as HSPA1A (HSP70), HSPA8 and HSP90AA1, together with OVGP1, gelsolin and ezrin, have important roles in the sperm-oviduct interaction and early embryo development, as we discussed in detail in our previous study [9]. Annexins 1, 2, 4, 5 which have been proposed as oviductal receptors for bovine sperm surface proteins and thus, serving to hold bovine sperm in the oviductal reservoir [40], were also found in oEVs. Clusterin (CLU) was increased during the postovulatory stage. Clusterin concentration in seminal plasma has been shown to correlate with sperm parameters such as reduced DNA fragmentation and sperm morphology [41] suggesting a positive effect of EVs’ CLU on gametes and the early embryo. Furthermore, CD109, which plays a role in avoiding detection by the female reproductive tract immune system [42], was found highly abundant in EVs and with no differential variation among stages.

### RNA cargo in oviductal EVs is under hormonal regulation

Total RNA isolated from oEVs with standard RNeasy micro kit and with miRNeasy mini kit (enriches shorter RNAs), respectively, were analyzed for size distribution and quality using the Agilent Bioanalyzer. Results from the RNA 6000 Pico chip for the RNeasy kit showed high integrity of the isolated RNA for most of the samples, with prominent 18S and 28S rRNA fragments (represented by peaks around 2000 and 4000 nt). Results from the small RNA assay (for miRNeasy) showed small RNA profiles in the range of less than 20 to 200 nucleotides, that could correspond to miRNAs (≈ 22 nt), tRNAs (≈ 60–90 nt), (snoRNA (≈ 70–90 nt), vault RNA (≈ 80–110 nt), Y_RNA (84–113 nt), snRNAs (≈ 63–190 nt), and small ribosomal RNAs (5S and 5.8 S). Results from mRNA-seq and small RNA-seq confirmed the presence of a high number of mRNAs and also different types of small ncRNA in oEVs as well as fragments of rRNAs and tRNAs, in line with the EVs RNA cargo derived from other sources [43, 44]. These studies showed that depending on the type of vesicles, intact protein-coding transcripts are present as well as ribosomal RNAs, and a variety of other small and long ncRNAs.

Regarding the mRNA content, we would like to highlight the presence of a high number of transcripts but in low copy number in oEVs. Only the top 500 most abundant transcripts showed a frequency of more than 25 transcripts per million. This arbitrary threshold was set to select transcripts for further functional analysis since we hypothesize that transcripts with lower concentrations in EVs are not very likely to have an effect on spermatozoa/embryo. Studies regarding the abundance and length of mRNAs loaded into EVs are rare and contradictory. Some studies found that EVs contained mainly RNA fragments [45]. In contrast, a few studies have demonstrated the presence of full-length and functional mRNA in exosomes [1]. Based on the results from the Bioanalyzer and the obtained mRNA-sequencing data, we conclude that bovine oEVs isolated with the protocol used in our study are containing full-length mRNAs and ncRNAs. The profile of the total RNA looked similar to total RNA isolated from cells or tissues. Furthermore, the analysis of exon coverage of the RNA-seq data (Additional file 2: Figure S12, IGV Sashimi plots) suggested the presence of complete undegraded transcripts. In addition, this analysis for some selected genes suggested the existence of splicing variants of mRNAs identified in oEVs.

Functional annotation revealed clusters of related terms with highest enrichments scores for the 500 most abundant transcripts in S1, were associated to protein translation and transport, extracellular vesicles, gene expression, response to stress and immune response and response to steroid hormones. We hypothesized that these transcripts could be transferred to gametes/embryos, in which they could be translated to functional proteins and exert an effect.

Our data also showed that mRNAs encoding ribosomal proteins of the large (RPL) and small subunit (RPS), are the most abundant protein-coding RNAs in oEVs, which is in line with other studies [43, 46]. Among the 100 most abundant mRNAs, we found 74 encoding ribosomal proteins and five mRNAs for translation elongation and initiation factors. Ribosomal proteins are playing an essential role in protein translation, but as well as, in tumorigenesis, immune signaling, and development [47]. Following our hypothesis that EVs mRNAs could be translated into proteins upon arrival to embryos, these transcripts may support ribosomal functions to ensure efficient translation of other EVs mRNAs where the EVs cargo is released [48]. It could even be possible that necessary components for protein translation are coming with the oEVs to the recipient cells since they contain rRNAs, ribosomal proteins, translation factors, and tRNAs. Among the most frequent transcripts were also mRNAs not involved in translation or protein processing. For the most frequent mRNA, tumor protein, translationally-controlled 1 (*TPT1*), has been shown to activate pluripotency genes such as *NANOG* and *OCT4* in oocytes [49].

In a similar way to the oEVs protein content, a strong difference in mRNA composition between S1 and the other stages was found. We identified a high number of differential transcripts (854) in oEVs between S1 vs S4. Our results are in line with Burns et al. [25], identifying an important number of differential transcripts (1611) in uterine EVs collected under different hormonal stimulus. Enriched GO terms for upregulated DET during S1 were mainly related to “extracellular exosome”, “extracellular vesicle”, and “oxidative phosphorylation”. While the downregulated DET were involved in embryo development, cell proliferation, epigenetic, regulation of histone modification and demethylation. Particularly interesting is the enrichment of functional categories related to epigenetic regulation of gene expression. These categories contained many genes involved in chromatin modification including histone methyltransferases (*EHMT1*, *EHMT2*, *EZH1*, *KMT2A*, *KMT2B*, *KMT2C*, *PHF1*, *PHF2*, *PRMT5*, *SETD1A*, *SETD2*) and histone demethylases (*ARID5B*, *JMJD1C*, *KDM2A*, *KDM3B*, *KDM5B*, *KDM5C*, *KDM6B*), but also one DNA methyltransferase gene (*DNMT1*), which were expressed in EVs of all stages but slightly lower in S1 EVs. This finding suggests that chromatin modification in the early embryo could be in part under maternal control via transcripts derived from oEVs. Furthermore, since studies in bovine IVP embryos showed distinct spatiotemporal expression patterns of genes involved in chromatin modification, a potential transfer of mRNAs of involved genes could be another factor for differences between in vitro and in vivo embryo development [50, 51] Considering how the hormones regulate the EVs RNA cargo, we pose the question whether the presence of spermatozoa and/or embryos in the oviduct could modulate the oEVs composition. Further investigations evaluating the oviductal response to sperm/embryo in terms of EVs release and cargo will help to answer this question.

Results from small RNA-sequencing revealed that a substantial proportion (76%) of reads aligned to rRNA in oEVs. A similar small ncRNA exosomal profile was observed in urinary EVs or EVs derived from human glioma stem cells culture among others, where rRNA fragments were also highly abundant [46, 52]. It has been suggested that the depletion of rRNA in oEVs may have increased the identification of a higher amount of other types of small ncRNAs (sncRNAs) [52]. However, many rRNA fragments and fragments of other non-miRNA ncRNA were in the size range of miRNAs and thus difficult to remove from miRNA sequences. Furthermore, pre-miRNAs would be removed by using size selection of the library products.

In contrast to the protein and mRNA content, the sncRNA content did not show a clear grouping of stages based on all sncRNA types or the individual RNA types analyzed (rRNA, tRNA, snoRNA, miRNA and other ncRNA). Only for snRNA, a partial grouping of S1-S2 and S3-S4 samples was observed. Despite no clear separation, higher differences in sncRNA were also found between S1 and S4 than between the rest of stages analyzed.

Regarding the miRNA content in oEVs, miRNAs represented with 11–18% of total read counts in the small RNA data set the most abundant RNA type after the fragments derived from rRNAs and tRNAs. Their importance as regulators of gene expression is well known as well as their important role in embryo development and embryo-maternal communication [53]. To date, dynamic changes of miRNA expression have been shown in both gametes and early embryos in different mammalian species [54–56]. Here, we demonstrated that oEVs miRNA content, which may be transferred to both gametes and embryos during the early reproductive events, is also dynamic and slightly under hormonal regulation. Burns et al.*,* [26] found that cyclic and pregnant uterine EVs contain also differential miRNA cargo, identifying only 1 miRNA specific for pregnant ewes, 27 miRNAs in cyclic and 53 in common. Our study shared 4 miRNAs with the Burns et al. [26] data (bta-miR-423 found in pregnant, bta-miR-429 in cyclic ewes, and bta-miR-148a, hsa-miR-151 in common).

Among the most abundant miRNAs in oEVs were several members of the let-7 family (4 under the top 10), which appear in exosomes from other cell/tissues and has been shown to have a conserved role in cell fate determination in the early embryo [57]. Additional miRNAs involved in this process are miR-125b [58] and miR-375 [59] which were under the 30 most abundant miRNAs in oEVs. Furthermore, increased expression of miR-148a, the sixth most frequent miRNA in oEVs, has been shown to improve early development of porcine cloned embryos by affecting DNA methylation via its target *DNMT1* [60].

Other interesting but less studied sncRNAs such as: tRNA fragments (representing 5% of sncRNA content) and Y-RNA (representing the 4% of other sncRNA) were also identified in oEVs. Transfer RNA fragments have been associated with a number of regulatory processes, e.g., during posttesticular sperm maturation and also regulation of endogenous retroelements active in preimplantation embryos [61]. Y-RNA was found in EVs in 3 biotypes RNY1, RNY3, RNY4, being the RNY4 the most abundant. Y-RNAs function for example at the initiation step of mammalian chromosomal DNA replication [62] and RNA quality control via binding to RO60 protein [63]. Both tRNA fragments and Y RNAs were also identified in exosomes in human semen by Vojtech et al.*,* [64]. These authors suggested they could play immunomodulatory functions in the female tract by the delivery of these small RNA molecules in exosomal semen. Based on the current knowledge about these sncRNAs, we hypothesize that tRNAs and Y-RNAs packed in oEVs could be involved in the immunoregulation of the oviduct epithelium in preparation for the arrival of spermatozoa and/or the embryo, contributing to maternal-sperm/embryo tolerance in the oviduct or maybe are transferred to the early embryo to assist processes involved in genome replication and activation. Among other sncRNA types identified in oEVs, we found snRNAs (e.g., RN7SK, spliceosomal RNAs) which were the only sncRNAs for which a grouping of stages was found in the PCA analysis based on their expression levels. These snRNAs have been associated to regulation of RNA biogenesis and RNA stability [65] as well as immune tolerance [66].

### Integration of oviductal EVs molecular content across the estrous cycle

In attempt to integrate the mRNA and protein data, we compared all transcripts and proteins identified in bovine oEVs across the estrus cycle, which revealed that almost all proteins (96%) were overlapping with the transcripts identified in oviductal EVs. Only 13 proteins were not found in all transcripts annotation such as hemoglobin, serotransferrin, albumin, apolipoprotein, which may be not contained in EVs from BOEC, but in EVs from blood or plasma. Besides, other proteins such as immunoglobulins, histone and other uncharacterized proteins were not identified among all transcripts, which may be due to annotation problems. However, here we found a very high overlap between transcripts and proteins identified in oEVs. Similar findings were obtained by Ogawa and colleagues [43], who suggested that protein-coding RNAs and expressed proteins may coexist in secreted EVs.

### Biological relevance of oviductal EVs’ content on sperm/embryo: Implications for the reproductive success

EVs represent ideal natural nanoshuttles for carrying specific molecules into spermatozoa, the oocyte and/or the embryo that are not present in the in vitro culture system. Moreover, the oEVs cargo is a “cocktail” of transcripts, proteins, miRNAs and lipids that can be used as a strategy to overcome the in vitro culture deficiencies and promote successful pregnancy. The transfer of proteins from oviduct to sperm/embryos via EVs can have a direct impact on sperm capacitation and fertilization ability [11, 24] or supporting early embryo development. The transfer of mRNA content could lead to the generation of functional proteins by the embryo and enabling horizontal transfer of genetic information. The transfer of miRNA content to sperm/embryos could have a remarkable impact on regulation of gene expression [12]. Recently, we demonstrated [9] that oEVs supplementation during in vitro culture improved embryo development, quality and survival in vitro. Furthermore, Lopera-Vásquez et al. [8, 23] demonstrated that oEVs also improve embryo cryosurvival and alter embryonic gene expression. Therefore, deciphering the molecular cargo of oEVs that could be responsible for the increase in embryo yield as well as in embryo quality and also, their potential impact on spermatozoa during pre- and post- fertilization periods seems imperative for us.

Comparative studies regarding the quality of embryos derived from ART versus in vivo embryos have shown important differences in the embryonic transcriptome and proteome profiles. Corcoran et al.*,* [67] showed suppressed expression of genes involved in transcription and translation (*CNOT1*, *EEF1G*, *PABPN1*, *FOXO3A*, *DOT1L*) and metabolism (*GALE*, *HEBP1*) in in vitro bovine embryos when compared with in vivo ones. These transcripts were identified in oEVs in our study. At the proteomic level, a marked reduced expression of annexins 1 and 2 in nuclear transfer (NT) derived embryos was observed and associated to the frequent failures of NT pregnancies, since annexins 1 and 2 are abundant in the placenta and have functions important to the maintenance of placentation [68]. Annexin 1 and 2 were among the most abundant proteins in oEVs. Considering these findings, the immediate question that arises is whether the supplementation of in vitro culture media with oEVs could restore the transcript and protein abundance to the in vivo embryos levels and thus improving the embryo quality. In this regard, Lopera-Vásquez et al.*,* [8] showed an upregulated expression of connexin 43 (also known as *GJA1*) and *GAPDH* in in vitro produced embryos after EVs supplementation during culture. Both transcripts are associated with better quality embryos and increased cryotolerance [69] and were also identified in the present study. Moreover, GAPDH plays an important role in energy metabolism and its mRNA was found as highly abundant in oEVs (among the 100 most abundant).

On the sperm side, the transcript abundance also varies with differences in sperm motility and is altered after sperm cryopreservation [70]. Cation channels CATSPER are very important for sperm fertility. The mRNAs for the sperm Ca^2+^ channel auxiliary subunits CATSPERγ and CATSPERδ were also identifed in oEVs. The encoded proteins are required for CATSPER channel function [71]. Cold inducible RNA binding protein (*CIRBP*) mRNA was present in oEVs and has been found as decreased in bovine frozen sperm [72] . The known and putative functions of many transcripts identified in oEVs led us to suggest whether the pre-incubation of sperm in diluents supplemented with EVs, during fertilization treatments or after cryopreservation/thawing, could increase the low abundance of specific transcripts in the spermatozoa and improve their functionality. Sperm can uptake EVs and transfer a specific protein (PMCA4) with an impact on sperm fertility, as demonstrated by Al-Dossary et al. [7]. The transfer of transcripts into sperm could have important implications in fertility, since some sperm RNAs are transferred into early embryos and regulate cell signaling processes or modulate epigenetic events during embryo development [70].

In this regard, EVs-associated miRNAs have been proposed as important players in fertilization and early embryonic development [53]. This is supported by the finding that many of the most abundant miRNAs identified in oEVs are known to be involved in the regulation of early embryo development and epigenetic regulation of gene expression (see above). With respect to miRNA expression regulation in oEVs during the estrous cycle, we focused on miR-449a, which was significantly upregulated in EVs during S1 compared to the rest of the analyzed stages, and has been associated to different causes of infertility in men and women. Dysregulation of miR-449a and miR-34c have been associated with defective cilia in the oviduct, inducing alterations in the transport of spermatozoa to meet the oocyte or the transport of the embryo to the uterus and thus, leading to infertility [73]. Furthermore, miR-449a has been associated with the regulation of multiciliogenesis [74]. In addition, a reduction of miR-34b-3p expression in human spermatozoa has been found to be directly related to aging [75].

Furthermore, target gene analysis of miR-449a showed a network of genes associated also to embryo development, angiogenesis, response to oxidative stress and chemotaxis or cell motility, suggesting implications in successful pregnancy. Moreover, hsa-miR-30d, identified also in our study and in EVs from human uterine fluid is taken up by mouse embryos, resulting in modifications of the embryonic transcriptome and embryo adhesion [12]. Altogether, this suggests that EVs’ miRNAs act as modulators of gene expression involved in development of the early embryo. Indeed, EVs associated RNAs have been proposed as a carrier of epigenetic information [76]. In this context, Reilly et al.*,* [13] demonstrated the selective transfer of miRNA cargo between epididymosomes and mouse spermatozoa. The transfer of miRNA via EVs could be a good strategy to increase the low number of specific miRNAs in subfertile sperm samples, modifying the sperm epigenome. Specific miRNAs were differentially represented in fertile versus subfertile cryopreserved bull semen [77] and low versus high motile bull cryopreserved sperm [78].

At this point, we would like to bring up that it is important to distinguish between the presence of specific transcripts, proteins, miRNA or other molecules in oEVs and their functional activity on the sperm/embryos. Currently, there is a dearth of knowledge of the actual activity levels of the transcripts, proteins or miRNAs found in EVs in the recipient cells, which it is even more unexplored in regard to reproductive processes. Further investigations are required to define precisely which components of the EVs cargo are most important and how the fate of spermatozoa and early embryos may be dictated by the EVs molecular cargo. Our study represents an essential starting point for: 1) understanding the potential role of oEVs in sperm functionality, fertilization and embryo development; 2) the design of more in-depth investigations about the functional activity of crucial molecules identified in oEVs on early sperm/embryo-maternal interactions and their potential use to improve the efficiency of different ART; 3) the development of synthetic EVs for the delivery of specific molecules into spermatozoa, embryos or the maternal tract, under different conditions of infertility.

## Conclusions

This is the first comprehensive attempt to reveal the mRNA, protein and small ncRNA content of oEVs. This study shows a complex and dynamic molecular signature of oEVs under the hormonal control of the estrous cycle, with marked differences between post-ovulatory and pre-ovulatory stages. Our findings reveal specific molecular cargo in oEVs with potential roles in sperm functionality, early embryonic gene expression regulation and development, and gamete/embryo-oviductal interactions. Our study provides a starting point for future more in-depth investigations of the role of EVs in early sperm/embryo-maternal interactions and their potential use to improve the efficiency of different reproductive biotechnologies.

## Methods

### Collection of samples

EVs samples were collected from oviducts under different hormonal regulation along the estrous cycle. Pairs of oviducts with their attached ovaries were collected from cyclic (corpus lutea (CL) present), nonpregnant cows at a local abattoir (Sablé sur Sarthe, France) and transported to the laboratory, with the permission of the direction of the slaughterhouse and the agreement of local sanitary services. Then, oviducts were classified into one of the 4 stages of the estrous cycle according to the morphology of the CL and follicle populations as previously described by Ireland et al.*,* [79] and used also by Lamy and collaborators [16] recently. Briefly, the stage of the estrous cycle classified as stage 1 (S1): showed recently ovulated follicle (d 1 to 4 of estrous cycle); stage 2 (S2): early luteal development with medium or large follicles or both present (d 5 to 11); stage 3 (S3): fully functional CL yellow or orange in color (d 11 to 17); or stage 4 (S4): regressing CL with little vasculature and a large preovulatory follicle present (d 18 to 20).

### Isolation of oviductal EVs

Oviducts were dissected free from surrounding tissues and flushed with 500 μl of sterile PBS (Sigma P4417-TAB, Lyon, France) to recover the oviductal fluid. EVs were isolated by ultracentrifugation [9, 80]. First, flushing samples were centrifuged at 300 *g* for 15 min, followed by 2000 *g* for 15 min, and then at 12000 *g* for 15 min to remove cells, blood, and cell debris and centrifuged twice at 100,000 *g* for 90 min (BECKMAN L8-M; SW41T1 rotor) to pellet exosomes. The pellets were resuspended in 50 μL of PBS and stored at − 80 °C for further analysis. A total of 60 animals were used in different 5 replicates for the 4 stages of the estrous cycle analyzed. Oviductal flushings from 3 animals were pooled for each replicate. Same EVs samples were used for transmission electron microscopy observations and proteomic and transcriptomic analysis.

### Characterization and quantification of oviductal EVs across the bovine estrus cycle

#### Transmission electron microscopy (TEM)

EVs suspensions were diluted in PBS to attain a protein concentration of 0.6 μg per μL and evaluated for size distribution by TEM. Three microliters of the sample were placed on the formvar carbon-coated grid for 5 min and washed with distilled water (three times). For negative contrast the samples were incubated in 2% water solution of uranyl acetate (30 s three times, 5 μl) and left to dry in the small drop (near 1 μl) of last solution. The micrographs were obtained using TEM HITACHI HT 7700 Elexience at 80 kV (with a charge-coupled device camera AMT) and JEM 1011 (JEOL, Japan) equipped with a Gatan digital camera driven by Digital Micrograph software (Gatan, Pleasanton, USA) at 100 kV. The processing of the photos and vesicle size calculation were carried out by ImageJ software. For TEM analysis, 3 different replicates of EVs samples at 4 different stages of the estrous cycle were analyzed.

#### Nanoparticle tracking analysis (NTA)

EVs suspensions were also evaluated for size and concentration on Nanosight equipment (Malvern, Inc.) using the software NTA 3.1 Build 3.1.45. The initial dilution factor used for the reading was 1:100 with PBS diluent. EVs analysis were performed with the sCMOS camera at the Camera Level of 13, in 5 videos of 30 s each and temperature controlled of 37 °C. For the analysis performed by the software Detect Threshold was 3 and Blur Size was Auto. The NTA analysis was performed in a different batch of samples (3 replicates/4 stages).

### RNA-sequencing and analysis

#### Messenger RNA (mRNA) library preparation, sequencing and data analysis

RNA was isolated from a total of 20 EVs samples (5 replicates, 4 stages) using RNeasy Micro kit (QIAGEN) according to the manufacturer’s instructions. RNA quality and concentration was evaluated by Agilent 2100 Bionalyzer (Agilent Technologies, Santa Clara, CA.) and NanoDrop (ThermoFisher). Poly(A) RNA was isolated starting from of 450 ng of total RNA by the use of NEXTflex™ Poly(A) beads. Poly(A) RNA was used for the preparation of mRNA libraries with the NEXTflex™ Rapid Directional qRNA-Seq Kit (Bioo Scientific). Library preparation followed the manufacturer’s instructions. Sequencing of the libraries was conducted on an Illumina HiSeq 2500 instrument at the Functional Genomics Center Zurich (FGCZ). Pooled barcoded libraries were run on two lanes of a single flow cell generating between 4 and 11 million single-end reads (125 bp) per sample.

The obtained sequence reads (Fastq files) were analyzed with an established analysis pipeline integrated in a local Galaxy installation [81] at the Animal Physiology group, ETH, Zurich. First, the sequence reads were processed with Trim Galore! to remove low quality from the 3′ end, adapter sequences, and sequences below a minimal length of 50 nt after the trimming steps. Subsequently, removal of PCR duplicates was done using FastUniq tool. Then, the tool Trimmomatic was used to cut 9 bases from the start of the read (HEADCROP) as these bases are derived from the molecular code contained in the adapter sequence. To control the performance of the processing steps, all Fastq files were quality checked after each processing step with FastQC (v0.11.2) and MultiQC. Reads were mapped with HISAT2 (v1.4.0) to the bovine genome sequence assembly (bosTau8, UMD_3.1.1). Based on the most current bovine GFF3 annotation file from NCBI and the mapping information for the reads (BAM files), a read count table for all annotated bovine mRNA sequences was generated using QuasR Qcount. This count table was filtered to remove sequences with negligible read counts by using counts per million (CPM) per sample filtering tool [82] (https://www.bioconductor.org/packages/devel/bioc/vignettes/edgeR/inst/doc/edgeRUsersGuide.pdf). The mean library size and potential CPM cutoff (Counttable statistics, in-house tool) was calculated and the cutoff set to 4.21 CPM (corresponding to an average of 20 reads per library) for at least 3 out of 20 libraries. This count table was the basis for the subsequent statistical analysis.

From a total of 20 samples in mRNA seq-dataset, 2 outliers (S1_R5; S4_R3) were removed because the low integrity of mRNA (RIN < 5) and other 2 outliers (S2_R4, S3:_R1) were removed because of the presence of high amount of universal adaptor after clipping. Then, the cutoff was set to 3.93 CPM and for at least 2 out of 16 libraries.

The analysis of differential mRNA expression was performed using BioConductor package EdgeR [83]. Data normalization was performed on library size (TMM normalization) [84] and with GLM robust (estimateGLMRobustDisp) [85] function. For comparison of the experimental groups, the following contrasts were set: S2 versus S1; S3 vs. S1 and S4 vs. S1. An adjusted *p*-value (false discovery rate, FDR) of 0.1% was used as threshold for significance of differentially abundant mRNA sequences.

RNA-Seq data have been deposited in NCBI’s Gene Expression Omnibus and are accessible through GEO SuperSeries GSE110444 (mRNA data: GSE110399 and ncRNA data: GSE110443).

Access SuperSeries RNA data: https://www.ncbi.nlm.nih.gov/geo/query/acc.cgi?acc=GSE110444

Access Subseries mRNA data: https://www.ncbi.nlm.nih.gov/geo/query/acc.cgi?acc=GSE110399

#### Small RNA library preparation, sequencing and data analysis

RNA was isolated from the same 20 EVs samples (5 replicates, 4 stages) but using miRNeasy Mini kit (QIAGEN) according to the manufacturer’s instructions. A total of 444 ng RNA was used for the preparation of each small RNA library using NEXTflex™ Small RNA-Seq Kit v3 (Bioo Scientific). Sequencing of the small RNA libraries was conducted on an Illumina HiSeq 2500 instrument at the FGCZ.

The small RNA sequence data analysis was processed using the same Galaxy pipeline at ETH, Zurich as mentioned above. First, adapter sequences were removed (3′ adapter sequence (TGGAATTCTCGGGTGCCAAGG) with the tool “clip adapter” (Version 1.0.1 FASTX-toolkit by Assaf Gordon). PCR duplicates were removed using the four random nucleotides on each side of the RNA fragment introduced with the adapters as molecular codes by running the tool “Collapse”. Then, the four random bases were removed from each side and “Collapse” was again used to obtain the unique sequences and the corresponding read counts corrected for PCR duplicates. Subsequently, a count table for all obtained unique sequences corresponding to a small ncRNA was generated using a series of further standard Galaxy tools as well as converting tools from the ToolShed (https://toolshed.g2.bx.psu.edu/). The resulting table contained the unique sequences and the number of reads per sample. This count table was filtered to remove sequences with negligible read counts, comprising sequencing errors or sequences with very low evidence for potential expression by using CPM per sample filtering tool [82] (https://www.bioconductor.org/packages/devel/bioc/vignettes/edgeR/inst/doc/edgeRUsersGuide.pdf.) The minimum library size and potential CPM cutoff (Counttable statistics, in-house tool) was calculated and the cutoff set to 8.41 CPM (corresponding to an average of 20 reads per library) for at least 3 out of 20 libraries. This count table was the basis for the subsequent statistical analysis and to annotate the sequences.

From a total of 20 samples in small RNA seq-dataset, 2 outliers (S2_R1; S4_R5) were removed because of the low integrity of mRNA (RIN) and the presence of high amount of universal adaptor after clipping). Then, the cutoff was set to 8.41 CPM and for at least 3 out of 18 libraries.

Small RNA sequences were annotated based on BLAST (Basic Local Alignment Search Tool) searches against a number of sequence databases. The collection of BLAST databases contained sequences from miRBase (bovine, human, and porcine precursor and canonical mature miRNAs), transcript sequences from NCBI and Ensembl, including non-coding RNAs, as well as tRNA and piRNA cluster sequences retrieved from the NCBI *Bos taurus* UMD 3.1.1 GFF3 file and http://www.smallrnagroup.uni-mainz.de/piRNAclusterDB.html, Rosenkranz D. piRNA cluster database: a web resource for piRNA producing loci. Nucleic Acids Research 2016 44(D1):D223-D230, respectively. The current miRBase release 21 contains 808 precursors and 793 mature bovine miRNAs.

The analysis of differential abundance of sequences was performed using BioConductor package EdgeR [83]. To normalize the read count data, the dataset was processed with TMM normalization [84] and GLM robust (estimateGLMRobustDisp) [85]. For comparison of the experimental groups, the following contrasts were set: S2 versus S1; S3 vs. S1, and S4 vs. S1. An adjusted *p*-value (false discovery rate, FDR) of 5% was used as threshold for significance of differentially expressed sequences (separately for ncRNA, rRNA, snoRNA, snRNA, tRNA, and miRNA sequences). Expression values (counts per million, cpm) of identified differentially expressed sequences (DES) were further processed in R to perform hierarchical cluster analysis by samples and DES [Gregory R. Warnes et al., https://CRAN.R-project.org/package=gplots 2016].

Small RNA-Seq data have been deposited in NCBI’s Gene Expression Omnibus and are accessible through GEO SuperSeries GSE110444 (mRNA data: GSE110399 and ncRNA data: GSE110443).

Access SuperSeries RNA data: https://www.ncbi.nlm.nih.gov/geo/query/acc.cgi?acc=GSE110444

Access Subseries ncRNA data: https://www.ncbi.nlm.nih.gov/geo/query/acc.cgi?acc=GSE110443

### Proteomic analysis

EVs samples were analyzed by SDS-PAGE combined with nanoLiquid Chromatography coupled to high resolution tandem mass spectrometry (nanoLC-MS/MS) with spectral counting quantitative method.

#### Sample preparation for MS analysis

Protein concentrations in the EVs samples were determined using the Uptima BC Assay kit (Interchim, Montluçon, France) according to manufacturer’s instructions and using bovine serum albumin as a standard. EVs samples from replicate 1–4 from same stage were pooled (10 μg EVs preparation/replicate) and 4 pools corresponding to S1, S2, S3, and S4 samples were analyzed by spectrometry analysis. A total of 30 μg of total protein/stage of EVs pooled samples were used. EVs samples were suspended in Laemmli buffer [86], followed by vortexing and incubation in a water bath at 95C° 5 min. Then, EVs samples were included in 10% SDS-PAGE (8.3 cm × 7.3 cm × 1.5 mm gels, 50 V, 30 min) without fractionation. Thus, samples were concentrated in a single narrow band. The resulting protein bands from 4 stages (1 band/stage) were stained with Coomassie blue (G-250). Densitometric quantification of Coomassie blue-stained protein bands was performed by transmission acquisition with an ImageScanner (GE Healthcare, Orsay, France) and analyzed with TotalLab (Nonlinear Dynamics Limited, Newcastle, UK) to check for the equivalent amount of protein among samples. Then, each lane was cut horizontally in 3 bands for a quantitative proteomic analysis. Gel slices from the four pooled samples were in-gel digested by trypsin and peptides were analyzed by Nano LC-MS/MS as previously described [9].

#### Nano LC-MS/MS analysis

Peptide mixtures were analyzed by nanoflow liquid chromatography-tandem mass spectrometry (nanoLC–MS/MS). All experiments were performed on a LTQ Orbitrap Velos mass spectrometer (Thermo Fisher Scientific, Bremen, Germany) coupled to an Ultimate® 3000 RSLC Ultra High Pressure Liquid Chromatographer (Dionex, Amsterdam, The Netherlands) controlled by Chromeleon Software (version 6.8 SR11; Dionex, Amsterdam, The Netherlands). Six microliters of each sample were loaded on trap column for desalting and separated using nano-column as previously described by Labas et al. [87]. Three nanoLC-MS/MS analyses were performed for each in vivo and in vitro EVs preparations. Data were acquired using Xcalibur software (version 2.1; Thermo Fisher Scientific, San Jose, CA). The instrument was operated in positive data-dependent mode. Resolution in the Orbitrap was set to *R* = 60,000. In the scan range of m/z 300–1800, the 20 most intense peptide ions with charge states ≥2 were sequentially isolated (isolation width, 2 m/z; 1 microscan) and fragmented using Collision Induced Dissociation (CID). The ion selection threshold was 500 counts for MS/MS, and the maximum allowed ion accumulation times were 200 ms for full scans and 50 ms for CID-MS/MS in the LTQ. Target ion quantity for FT full MS was 1e6 and for MS/MS it was 1e4. The resulting fragment ions were scanned at the “normal scan rate” with q = 0.25 activation and activation time of 10 ms. Dynamic exclusion was active during 30 s with a repeat count of 1. The lock mass was enabled for accurate mass measurements. Polydimethylcyclosiloxane (m/z, 445.1200025, (Si(CH_3_)_2_O)_6_) ions were used for internal recalibration of the mass spectra.

#### Data processing and statistical analysis

MS/MS ion searches were performed using Mascot search engine v 2.2 (Matrix Science, London, UK) via Proteome Discoverer 2.1 software (ThermoFisher Scientific, Bremen, Germany) against a locally maintained copy of NCBInr (571,109 Mammalia entries, download January 2016). The parameters used for database searches included trypsin as a protease with two missed cleavages allowed, and carbamidomethylcysteine, oxidation of methionine and N-terminal protein acetylation as variable modifications and peptide charge 2 and 3+. The tolerance of the ions was set at 5 ppm for parent and 0.8 Da for fragment ion matches. Mascot results obtained from the target and decoy databases searches were subjected to Scaffold 4 software (v 4.4.7, Proteome Software, Portland, USA) for validation. Peptide identifications were accepted if they could be established at over 95.0% probability as specified by the Peptide Prophet algorithm [88]. A false discovery rate was calculated as < 1% at the peptide or protein level.

For comparative analysis, we employed Scaffold Q+ software (version 4.4, Proteome Software, Portland, USA) for spectral counting label-free quantitative method with “Weighed Spectra” option on protein clusters. A t-test was performed to characterize changes between in vivo and in vitro EVs preparations. Differences were considered statistically significant for t-tests (phase of cycle comparison) at *p*-value< 0.05. Limits of an average normalized weighted spectra (NWS) of ≥5 and fold change/ratio of ≥2 were included to increase validity of any comparisons made.

MS data have been deposited to the ProteomeXchange Consortium via the PRIDE [89] partner repository with the dataset identifier PXD008851 and 10.6019/PXD008851.

### Data mining and bioinformatics analysis

Gene symbols and Entrez Gene IDs (bovine and putative human orthologs) were mapped for all transcripts, small RNA and protein identifications and analyzed using bioinformatics custom tools integrated in a local Galaxy [81] installation. Custom database tools (NCBI annotation mapper, Mammalian Ortholog and Annotation database, MOADb; Bick J, ETH Zurich, unpublished results 2017) were used to assign known or putative human orthologous genes. Human gene identifiers or symbols were used for subsequent functional annotation. Functional analysis of transcripts and proteins contained in EVs was performed using DAVID 6.8 Functional Annotation (https://david.ncifcrf.gov) [90, 91]. DIANA-miRPath v3.0 and TarBase v.7 was used for gene target analysis from candidate miRNA and further functional analysis of target genes and clustering. TarBase v.7 allowed us to select only validated target genes from selected miRNA for pathways analysis. Comparative enrichment clustering and network was performed using ToppCluster tool [92] (https://toppcluster.cchmc.org) for differentially abundant transcripts (DT) and proteins (DP) across the bovine estrous cycle.

#### Protein and miRNA validation

##### Western blotting

Proteins were separated by SDS-PAGE and transferred onto nitrocellulose membranes (GE Healthcare Life Sciences Whatman™). The membranes were washed in distilled water and blocked with Tris-buffered saline (TBS) containing Tween 20 (0.5% (*w*/*v*)), and supplemented with lyophilized low-fat milk (5% w/v) for 1 h at room temperature. The membranes were incubated with primary antibodies diluted in TBS-Tween containing low-fat milk (1% w/v) for 2 h at 37 °C with gently shaking. The primary antibodies used were: anti-heat shock protein 70 (HSP70; Stressgen, SPA-810); anti-annexin I (ANXA1, Santa Cruz Biotechnologies, sc11387); anti-Cluster of Differentiation 109 (CD109, Santa Cruz Biotechnology, sc98793); anti-heat shock protein A8 (HSPA8; bioss.com, bs-5117R)**;** anti-Myosin heavy chain 9 (MYH9 (H40), Santa Cruz Biotechnology, sc-98,978). After primary antibodies incubation, the membranes were washed with TBS with 0.5% Tween 20 and incubated overnight at 4 °C under agitation with secondary antibodies. The secondary antibodies used were: horseradish peroxidase (HRP)-anti-mouse (Sigma A4416) or anti-rabbit (Sigma A6154). Blots were developed using a mixture of two chemiluminescence substrates developing kit (GE Healthcare Amersham^TH^ ECL Select^TH^ Western blotting detection Reagent RPN2235 and Supersignal West Pico #34087 Chemiluminescent Substrate Thermo Scientific).

##### Real-time PCR expression analysis of miRNAs

Comparative expression of bta-miR-449a along the bovine estrus cycle was analyzed by qPCR, since its dysregulation has been associated with defective cilia in the oviduct and infertility. Using a new batch of EVs isolated from oviducts classified in the four stages of the estrous cycle (*n* = 12), total RNA, including microRNAs, was purified from isolated oviduct EVs using the miRNeasy Mini Kit (QIAGEN), according to the manufacturer’s instructions. Mature and precursors microRNAs, mRNAs and non-coding RNAs were transformed into cDNA by reverse transcription using the miScript II RT Kit (QIAGEN), according to the manufacturer’s instructions. A 10 μL reaction was performed for each sample, with 200 ng of total RNA. Reverse transcription was performed in a thermocycler (Life Technology), incubated at 37 °C for 60 min, followed by 95 °C for 5 min to inactivate a reverse transcriptase. Samples were stored at − 20 °C.

The expressions of bta-miR-449a was analyzed using the miScript SYBR® Green PCR Kit (QIAGEN) according to the manufacturer’s instructions. Primers sequences (5′ - 3′)) used for qPCR: for bta-miR-449a (TGGCAGTGTATTGTTAGCTGGT) and 3 endogenous (RNT43 snoRNA: CTTATTGACGGGCGGACAGAAAC; Hm / Ms. / Rt T1 snRNA: CGACTGCATAATTTGTGGTAGTGG and bta-miR-99b: CACCCGTAGAACCGACCTTGCG. The reactions were performed in duplicate in total volume of 6 μl, containing 2X QuantiTect SYBR Green PCR Master Mix, 10X miScript Universal Primer, 10X miScript Primer Assay, RNase free-water and 0.4 ng of cDNA. The RT-PCR was performed on the QuantStudio 6 Flex (Life Technology) equipment. Temperature and time conditions were 95 °C for 15 min for activation of HotStarTaq DNA Polymerase and 45 cycles at 94 °C for 15 s for denaturation, 55 °C for 30 s for annealing and 70 °C for 30 s for extension. Finally, the melting curve was performed. MicroRNAs were considered present at Ct values below 35 cycles and the amplification product was confirmed from the melting curve analysis.

## Additional files

Additional file 1: Proteomic and Transcriptomic data of oEVs content. Contains: Table S0. All transcripts data; Table S1. Differentially expressed transcripts (DET) S2 vs S1; Table S2. DET S3 vs S1; Table S3. DET S4 vs S1; Table S4. All DET (FDR 0.001); Table S5. Top 500 mRNA TPM; Table S6. All proteins MS data; Table S7. Differentially expressed proteins (DEP); Table S8. All proteins annotation; Table S9. Small ncRNA; Table S10. miRNA; Table S11. Overlap transcripts and proteins. (XLSX 7063 kb)

Additional file 2: Additional Figures of the manuscript. **Figure S1.** Transcripts per Million (TPM) distribution identified in oviduct EVs across the bovine estrous cycle. **Figure S2.** Comparisons of differential abundant transcripts (DT) among stages (cutoff FDR < 0.001). **Figure S3.** Comparative analysis of the small ncRNA across the bovine estrous cycle. **Figure S4.** Comparisons of differential small ncRNA among stages (cutoff FDR < 0.05). **Figure S5.** Comparisons of differential rRNA among stages (cutoff FDR < 0.05). **Figure S6.** Comparisons of differential miRNA among stages (cutoff FDR < 0.05). **Figure S7.** Comparisons of differential tRNA among stages (cutoff FDR < 0.05). **Figure S8.** Comparisons of differential snRNA among stages (cutoff FDR < 0.05). **Figure S9.** Comparisons of differential other ncRNA among stages (cutoff FDR < 0.05). **Figure S10.** Oviductal EVs RNA isolated by the use of miRNeasy kit. **Figure S11.** Oviductal EVs RNA isolated by the use of RNeasy micro kit. **Figure S12.** IGV Sashimiplots mRNA. (PDF 8718 kb)

Additional file 3: Functional analysis of proteins, transcripts, and miRNAs identified in oEVs. Contains: Table S0. Functional analysis (FA) of the 500 most abundant mRNA. **Table S1.** ToppCluster FA of mRNA. **Table S2.** FA of all proteins. **Table S3.** ToppCluster FA of proteins. **Table S4.** Network of proteins downregulated in S1_a; **Table S5.** Network of proteins upregulated in S1_bb’; **Table S6.** Network of proteins upregulated in S2_c; **Table S7.** Network of proteins upregulated in S3_d; **Table S8.** Network of proteins downregulated in S4_e; **Table S9.** Target gene and Pathways analysis of selected miRNA with differential expression and high abundance in EVs in Stage 1 and Stage 4; **Table S10.** Heatmap data representing 13 selected miRNAs and their associated pathways (XLSX 151 kb)
