# Supplementary material for: Deciphering the oviductal extracellular vesicles content across the estrous cycle: implications for the gametes-oviduct interactions and the environment of the potential embryo
Source: BMC Genomics. 2018 Aug 22;19:622. doi: 10.1186/s12864-018-4982-5 (PMC6103977; doi:10.1186/s12864-018-4982-5)
Supplement: Supplementary file 2 — Additional Figures of the manuscript. Figure S1. Transcripts per Million (TPM) distribution identified in oviduct EVs across the bovine estrous cycle. Figure S2. Comparisons of differential abundant transcripts (DT) among stages (cutoff FDR < 0.001). Figure S3. Comparative analysis of the small ncRNA across the bovine estrous cycle. Figure S4. Comparisons of differential small ncRNA among stages (cutoff FDR < 0.05). Figure S5. Comparisons of differential rRNA among stages (cutoff FDR < 0.05). Figure S6. Comparisons of differential miRNA among stages (cutoff FDR < 0.05). Figure S7. Comparisons of differential tRNA among stages (cutoff FDR < 0.05). Figure S8. Comparisons of differential snRNA among stages (cutoff FDR < 0.05). Figure S9. Comparisons of differential other ncRNA among stages (cutoff FDR < 0.05). Figure S10. Oviductal EVs RNA isolated by the use of miRNeasy kit. Figure S11. Oviductal EVs RNA isolated by the use of RNeasy micro kit. Figure S12. IGV Sashimiplots mRNA. (PDF 8718 kb) [file 12864_2018_4982_MOESM2_ESM.pdf]

Additional File 2: Supplementary figures of the manuscript

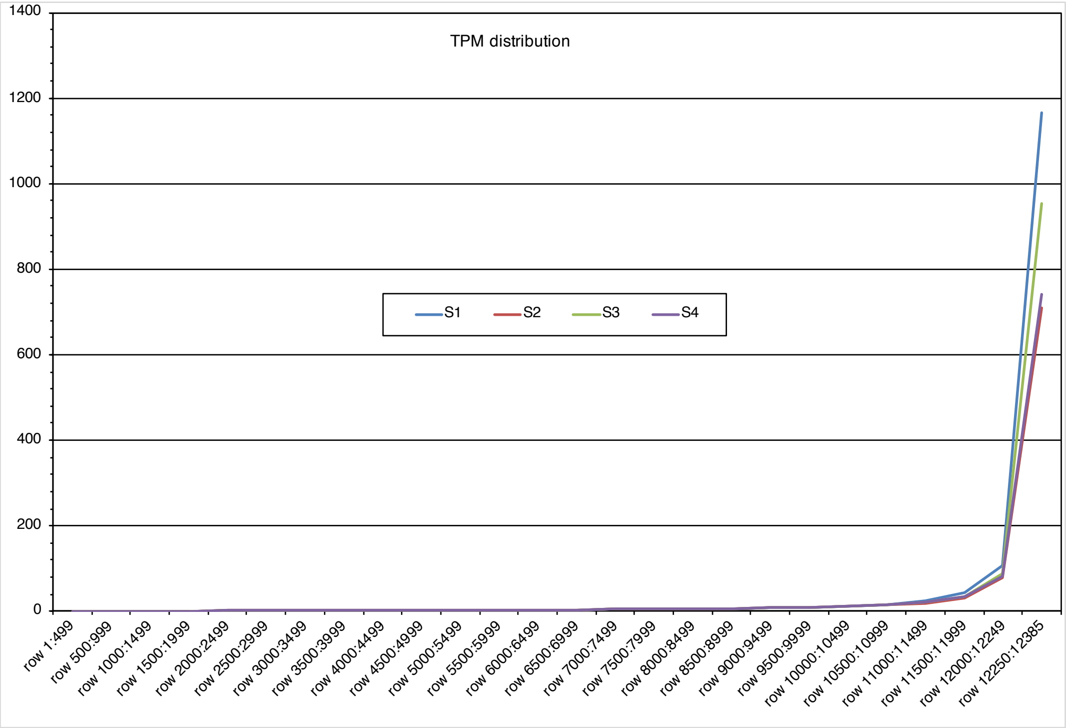

Figure S1. Transcripts per Million (TPM) distribution identified in oviduct EVs across the bovine estrous cycle.

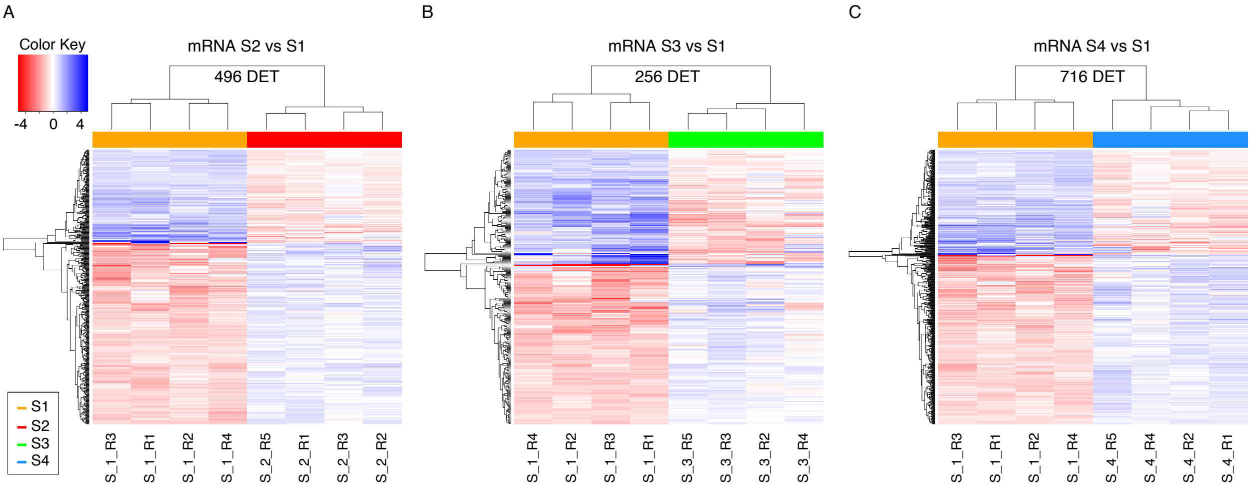

Figure S2. Comparisons of differential abundant transcripts (DT) among stages (FDR cut-off <0.001). Dendrograms representing results of unsupervised hierarchical clustering (HCL). Rows indicate single transcripts, while columns represent different stages of the estrous cycle compared. Hierarchical cluster analysis showed a clear separation between S2 and S1 (A), between S3 and S1 (B) and between S4 and S1 (C).

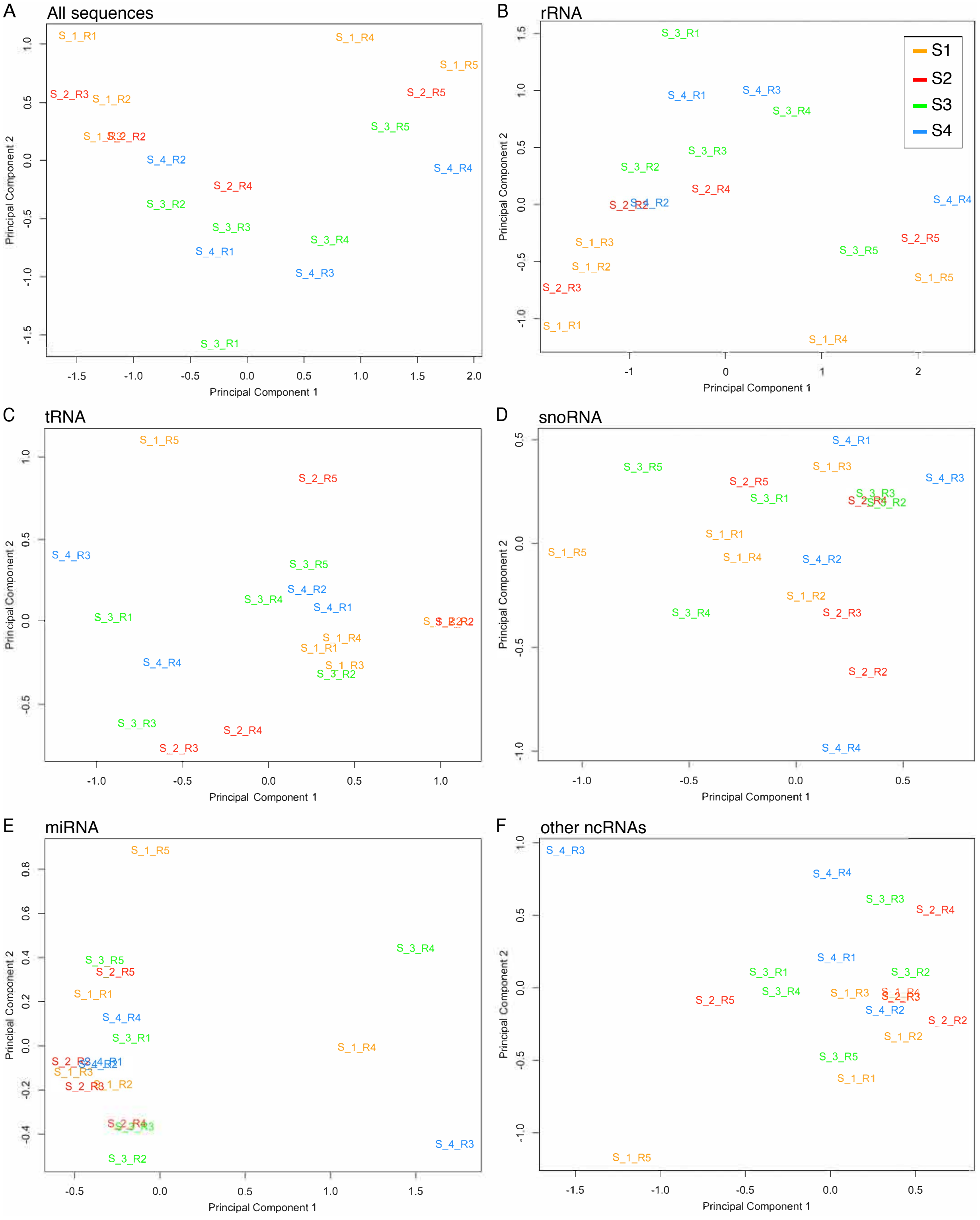

Figure S3. Comparative analysis of the small ncRNA across the bovine estrous cycle. Principal Component analysis (PCA) on small ncRNA oviduct EVs content at 4 different stages of the estrous cycle (S1 in yellow; S2 in red, S3 in green and S4 in blue) for the top 1000 small ncRNA for: **A)** All small ncRNA; **B)** ribosomal RNA (rRNA); **C)** transfer RNA (tRNA); **D)** small nucleolar RNA (snoRNA); **E)** microRNA (miRNA) and **F)** other ncRNA.

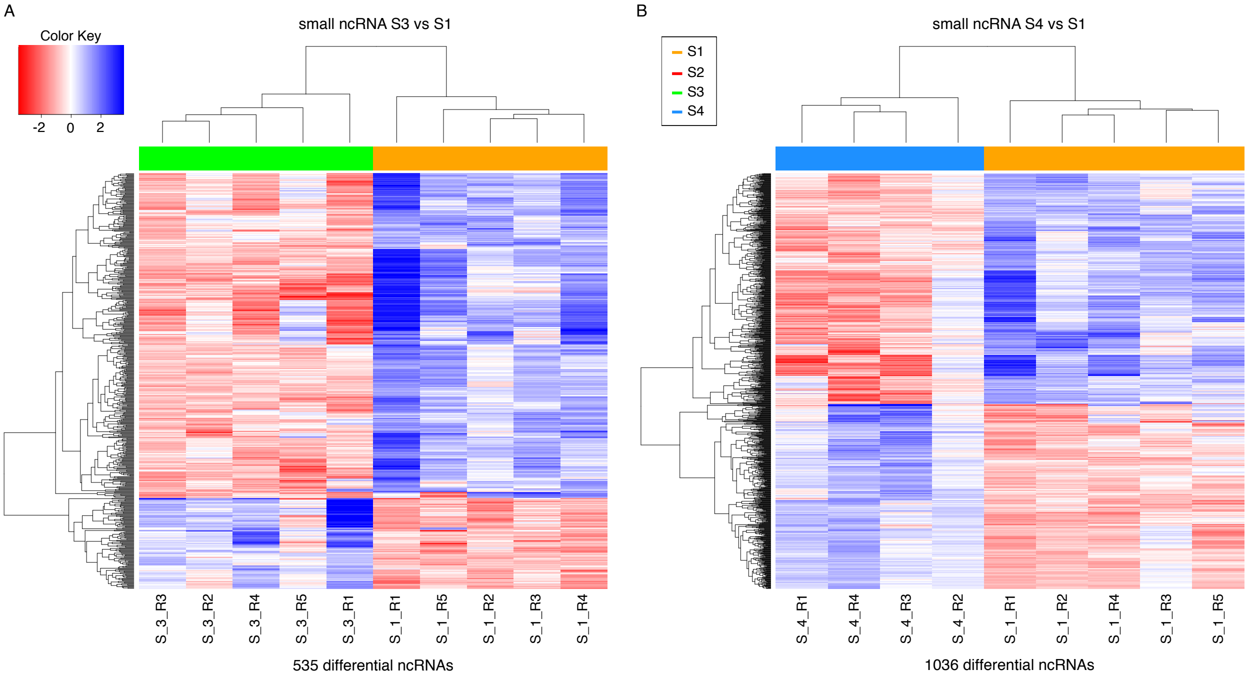

Figure S4. Comparisons of differential small ncRNA among stages (cut-off FDR < 0.05). Dendrograms representing results of unsupervised hierarchical clustering (HCL). Rows indicate single ncRNA, while columns represent different stages of the estrous cycle compared. Hierarchical cluster analysis showed a clear separation between S3 and S1 (A) and between S4 and S1 (B) for all small ncRNA.

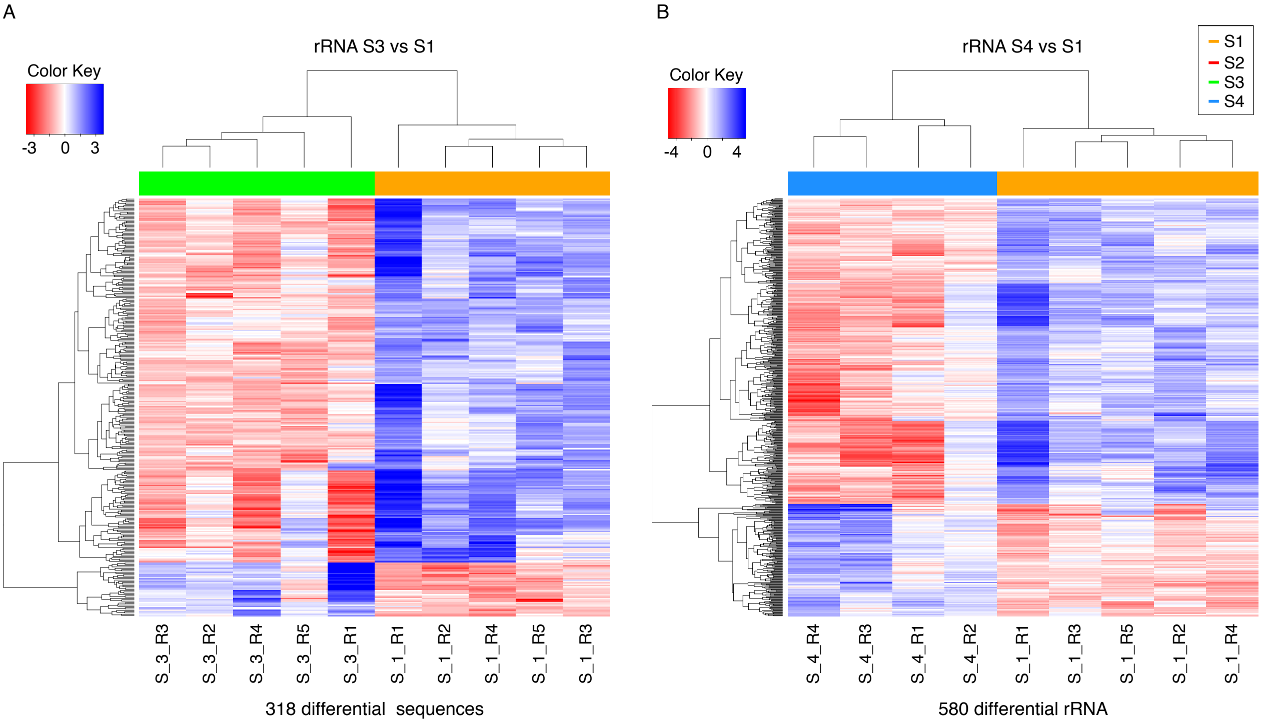

Figure S5. Comparisons of differential rRNA among stages (cut-off FDR < 0.05). Dendrograms representing results of unsupervised hierarchical clustering (HCL). Rows indicate single ncRNA, while columns represent different stages of the estrous cycle compared. Hierarchical cluster analysis showed a clear separation between S3 and S1 (A) and between S4 and S1 (B) for rRNA.

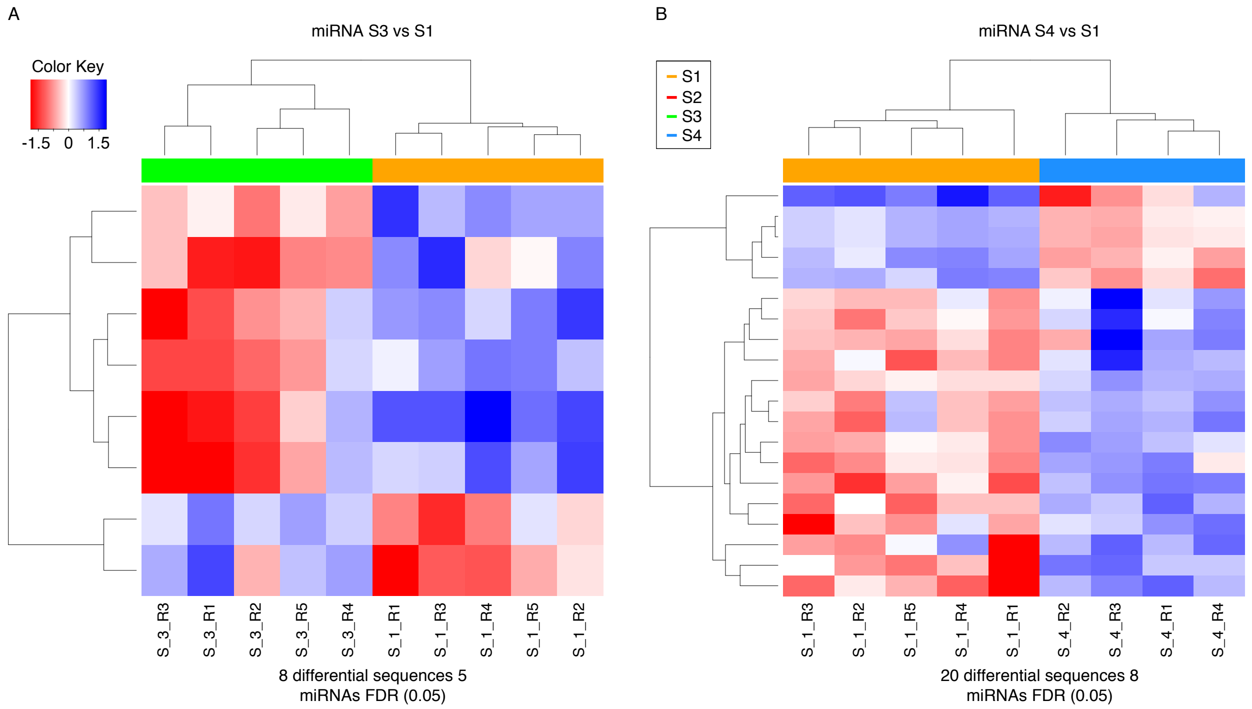

Figure S6. Comparisons of differential miRNA among stages (cut-off FDR < 0.05). Dendrograms representing results of unsupervised hierarchical clustering (HCL). Rows indicate single ncRNA, while columns represent different stages of the estrous cycle compared. Hierarchical cluster analysis showed a clear separation between S3 and S1 (A) and between S4 and S1 (B) for miRNA.

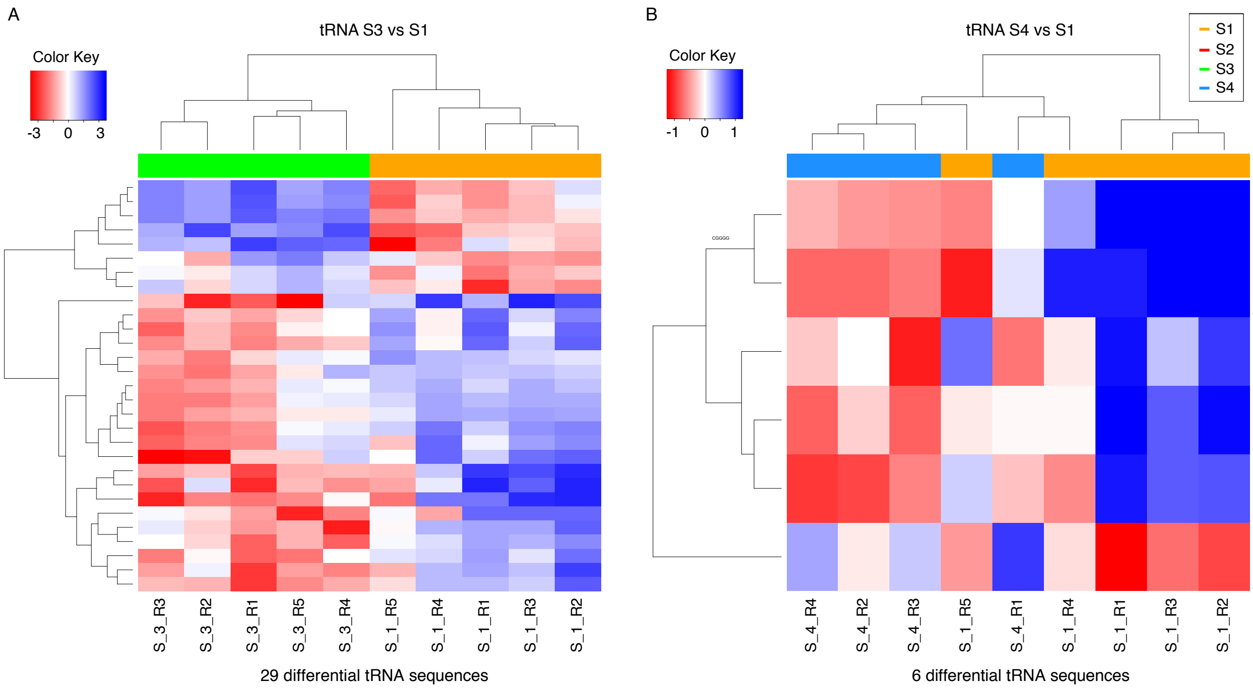

Figure S7. Comparisons of differential tRNA among stages (cut-off FDR < 0.05). Dendrograms representing results of unsupervised hierarchical clustering (HCL). Rows indicate single ncRNA, while columns represent different stages of the estrous cycle compared. Hierarchical cluster analysis showed a clear separation between S3 and S1 (A) and between S4 and S1 (B) for tRNA.

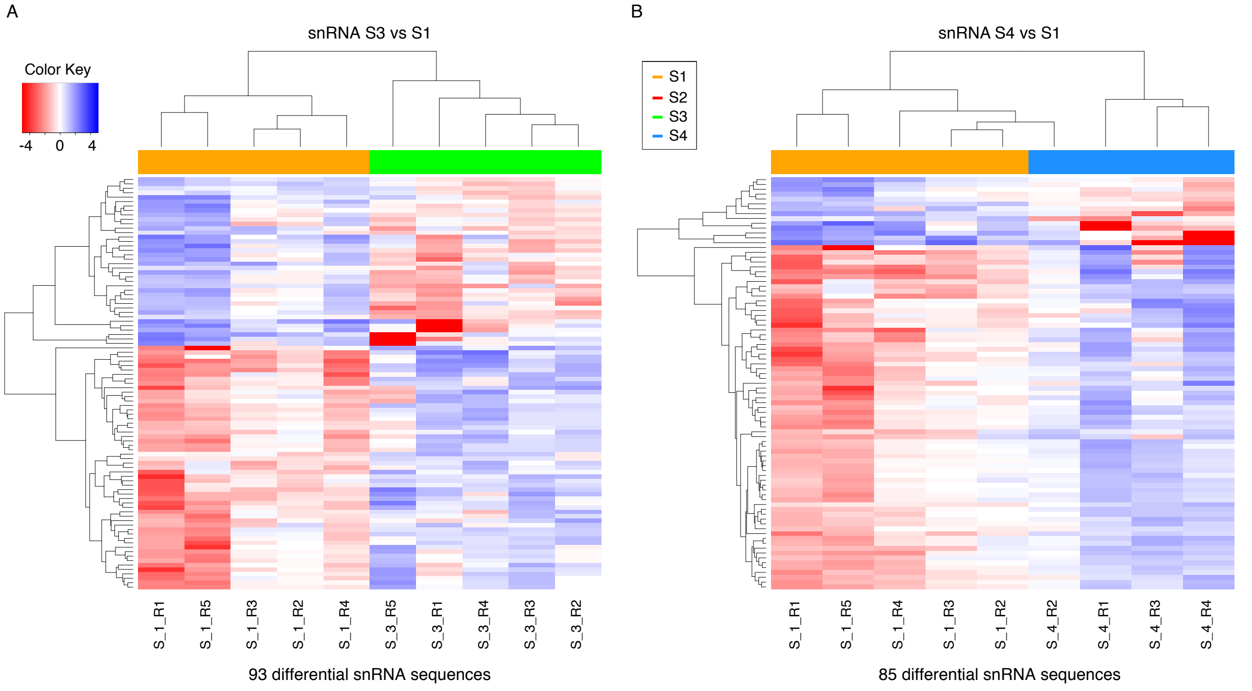

Figure S8. Comparisons of differential snRNA among stages (cut-off FDR < 0.05). Dendrograms representing results of unsupervised hierarchical clustering (HCL). Rows indicate

single ncRNA, while columns represent different stages of the estrous cycle compared. Hierarchical cluster analysis showed a clear separation between S3 and S1 (A) and between S4 and S1 (B) for snRNA.

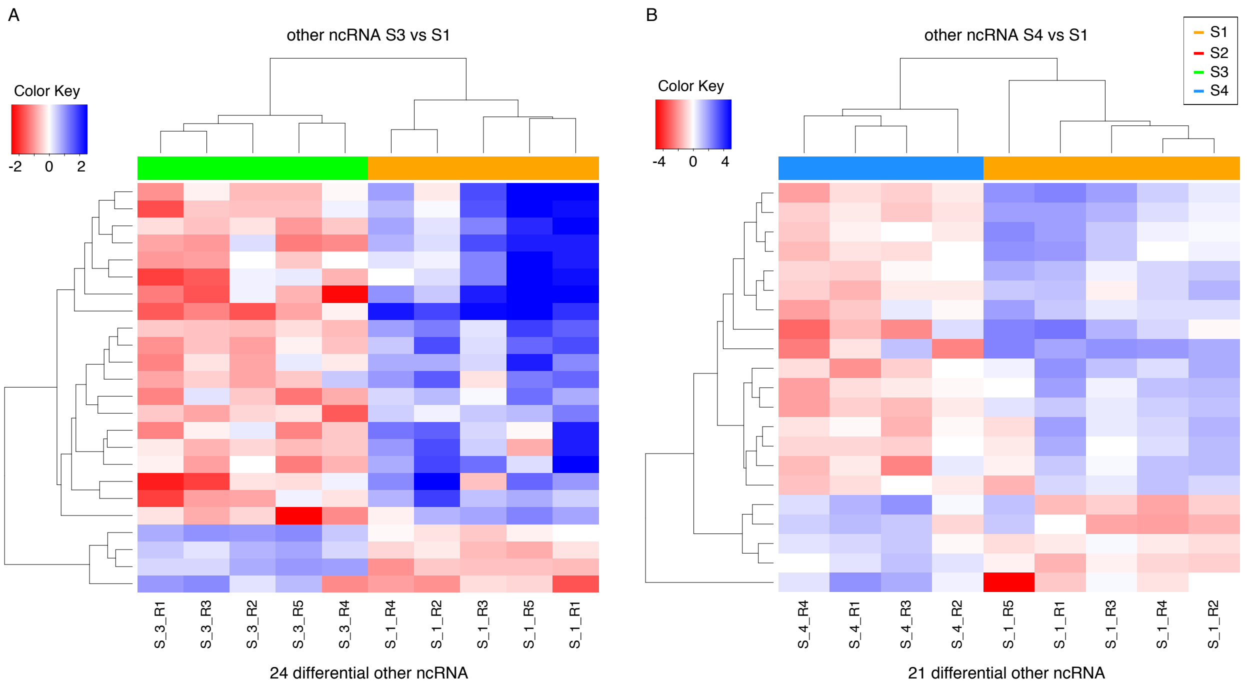

Figure S9. Comparisons of differential other ncRNA among stages (cut-off FDR < 0.05). Dendrograms representing results of unsupervised hierarchical clustering (HCL). Rows indicate single ncRNA, while columns represent different stages of the estrous cycle compared. Hierarchical cluster analysis showed a clear separation between S3 and S1 (A) and between S4 and S1 (B) for snRNA.

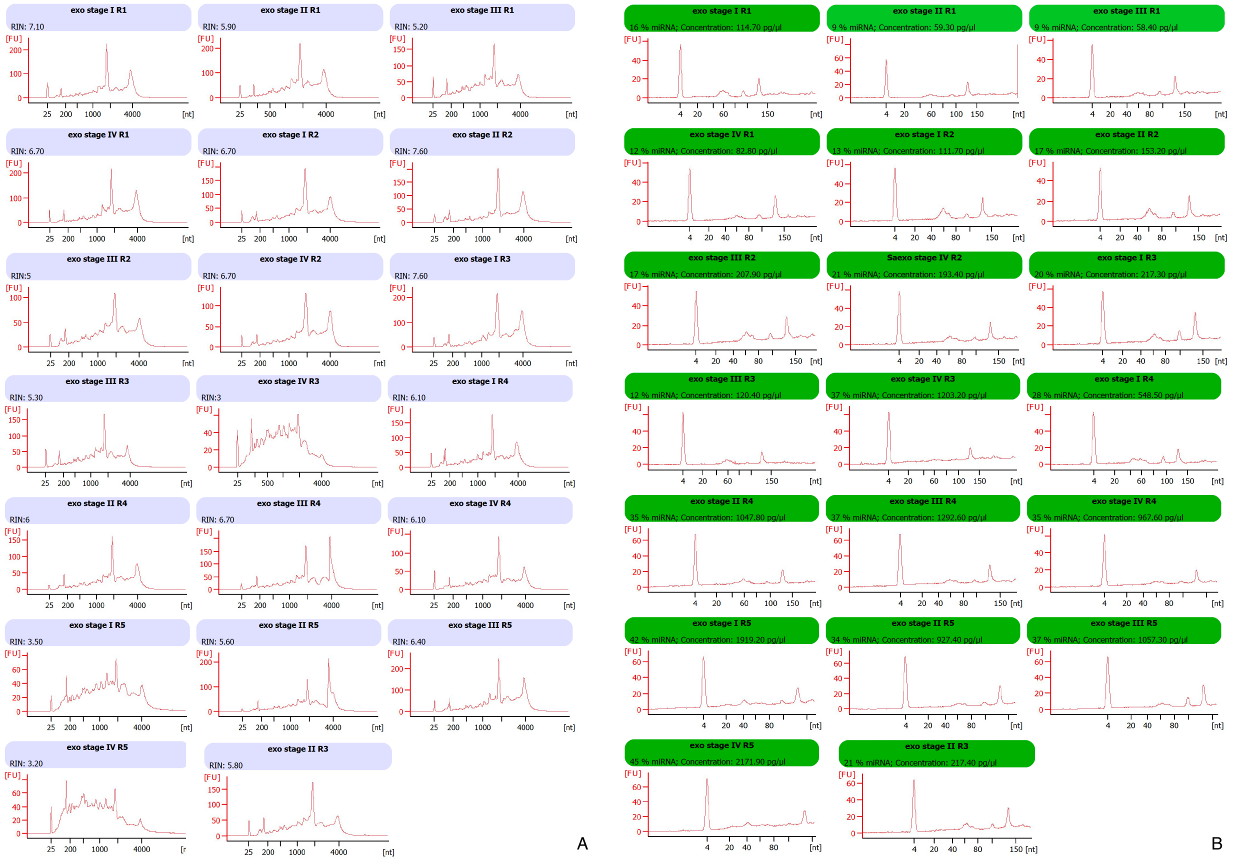

Figure S10. Oviductal EVs RNA isolated by the use of miRNeasy kit. RNA isolated with the miRNeasy kit (Qiagen) was analyzed using capillary electrophoresis with the Agilent RNA 6000 Pico chip (**A**) and the small RNA chip (**B**) on an Agilent 2100 Bioanalyzer ®. The y-axis of the electropherograms represents fluorescence units (FU) and the x-axis represents the nucleotide length of the RNA (nt). Peaks at 25 nt (Pico) and 4 nt (small RNA) represent the internal standard, respectively. Data shown represents the 20 samples analyzed for small ncRNA experiment. Results from the small RNA assay (for miRNeasy) showed small RNA profiles in the range of less than 20 to 200 nucleotides, that could correspond to miRNAs (≈ 22 nt), tRNAs (≈ 60-90 nt), (snoRNA (≈ 70-90 nt), vault RNA (≈ 80-110 nt), Y_RNA (84-113 nt), snRNAs (≈ 63-190 nt), and small ribosomal RNAs (5S and 5.8 S).

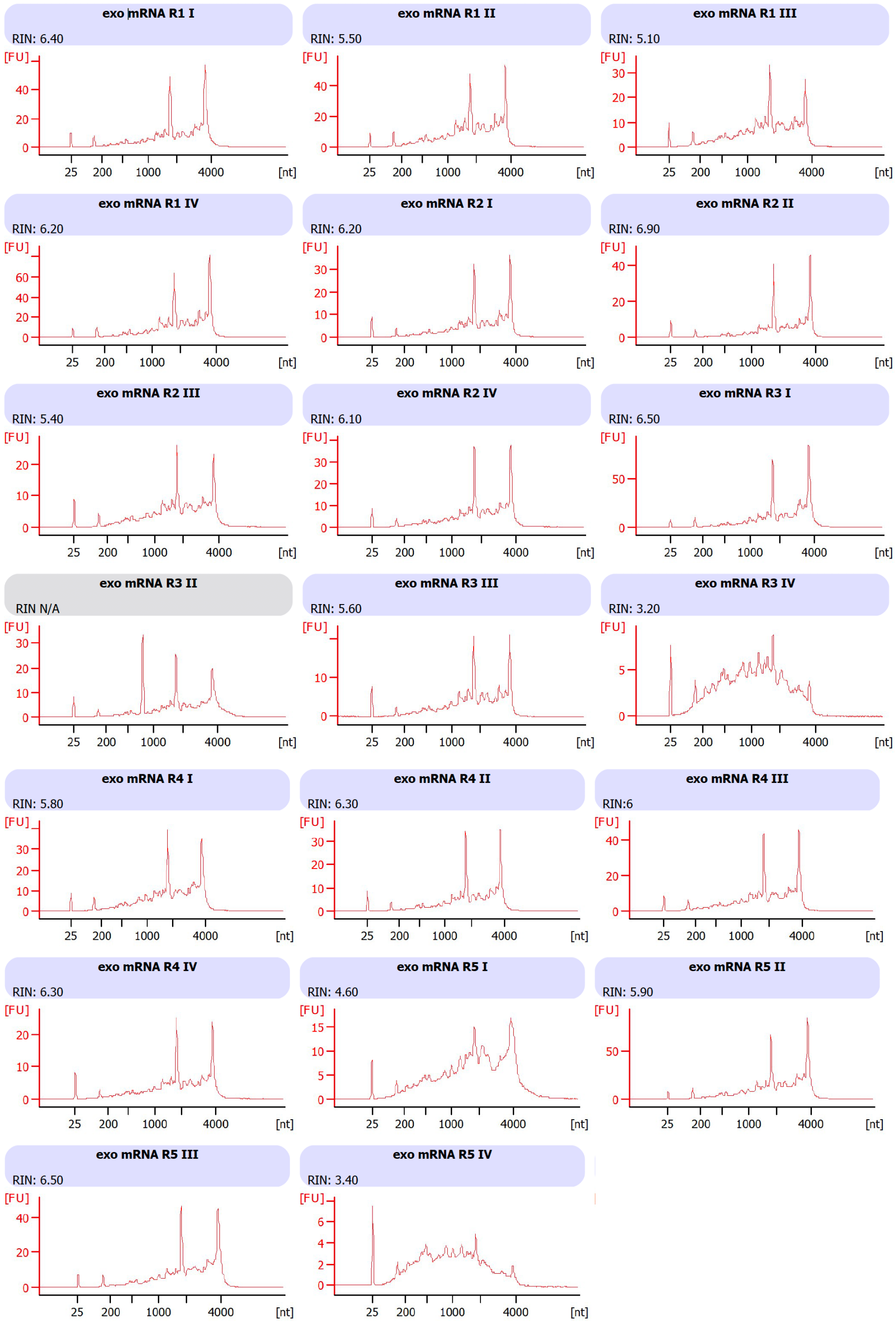

Figure S11.

Oviductal EVs RNA isolated by the use of RNeasy micro kit. RNA was analyzed using capillary electrophoresis with the Agilent RNA 6000 Pico chip on an Agilent 2100 Bioanalyzer ®. The y-axis of the electropherograms represents fluorescence units (FU) and the x-axis represents the nucleotide length of the RNA (nt). Peak at 25 nt represents the internal standard. Data shown represents the 20 samples analyzed for mRNA experiment. Results from Pico chip showed the high integrity of RNA, with prominent 18S and 28S rRNA peaks (represented by spikes around 2000 and 4000 nt).

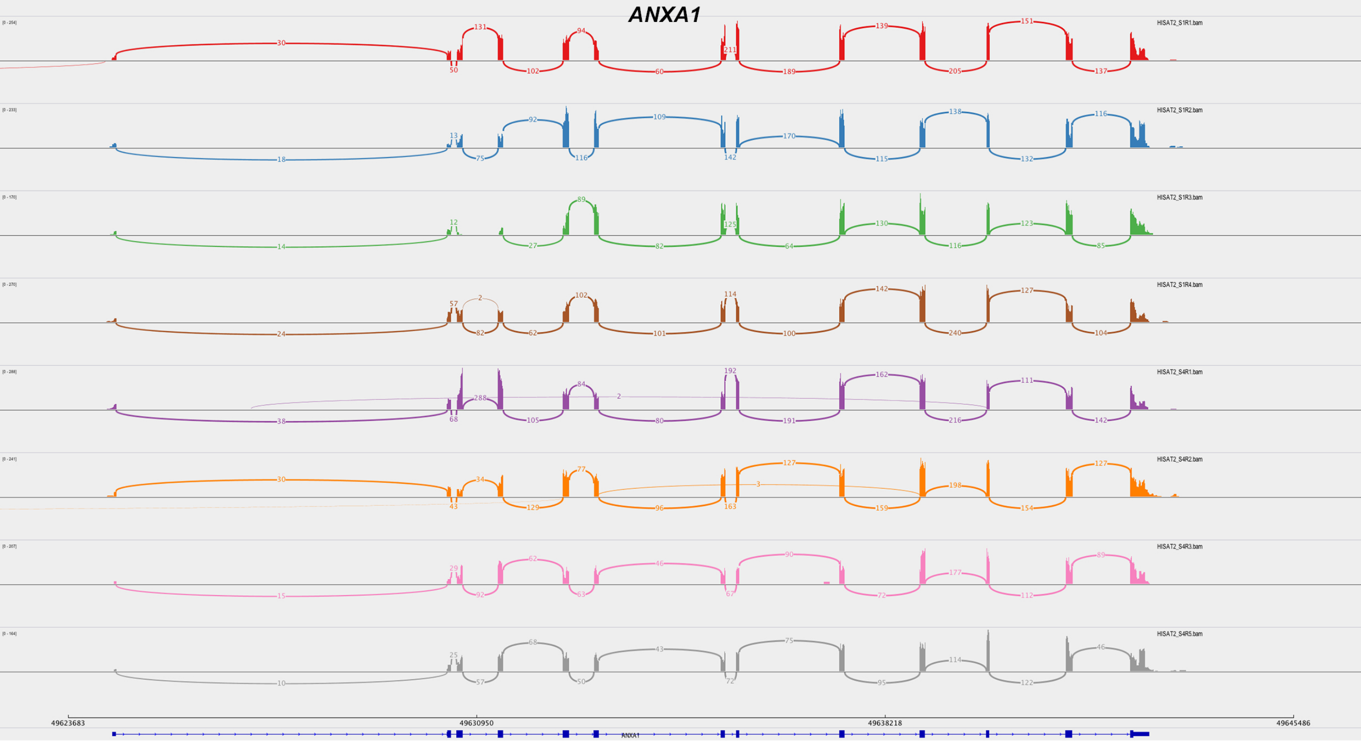

Figure S12.

Analysis of RNA-seq coverage and splice junctions. Integrated Genome Viewer (IGV, v.2.4.8) was used to analyze read coverage and spliced reads of a number of selected genes based on the BAM files for 4 replicates of stage 1 and stage 4, respectively. This and the followign panels show so-called Sashimi plots. At the bottom the annotated exons of the respective genes are shown in blue (exons=boxes, introns=lines, arrows=direction of the gene).

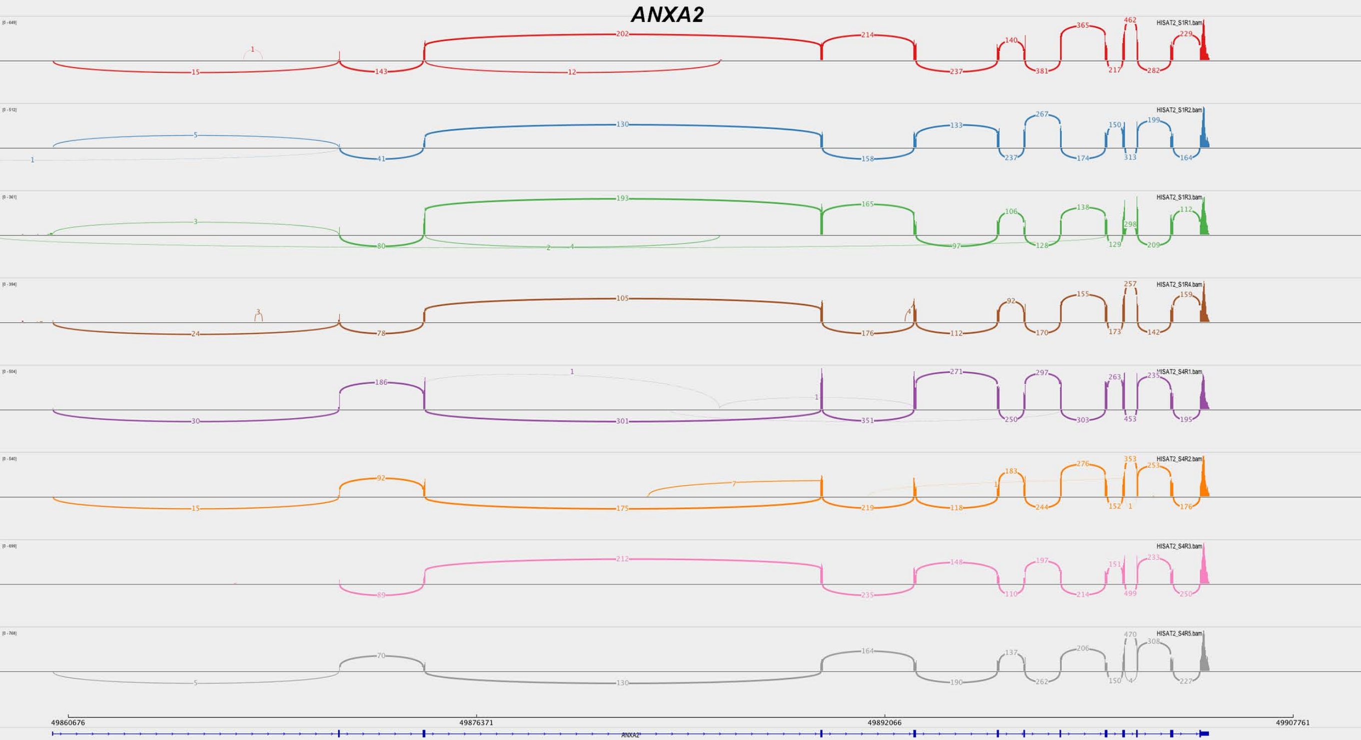
ANXA2
HISAT2_S1R1.bam
HISAT2_S1R2.bam
HISAT2_S1R3.bam
HISAT2_S1R4.bam
HISAT2_S4R1.bam
HISAT2_S4R2.bam
HISAT2_S4R3.bam
HISAT2_S4R5.bam
49860676
49876371
49892066
49907761
ANXA2

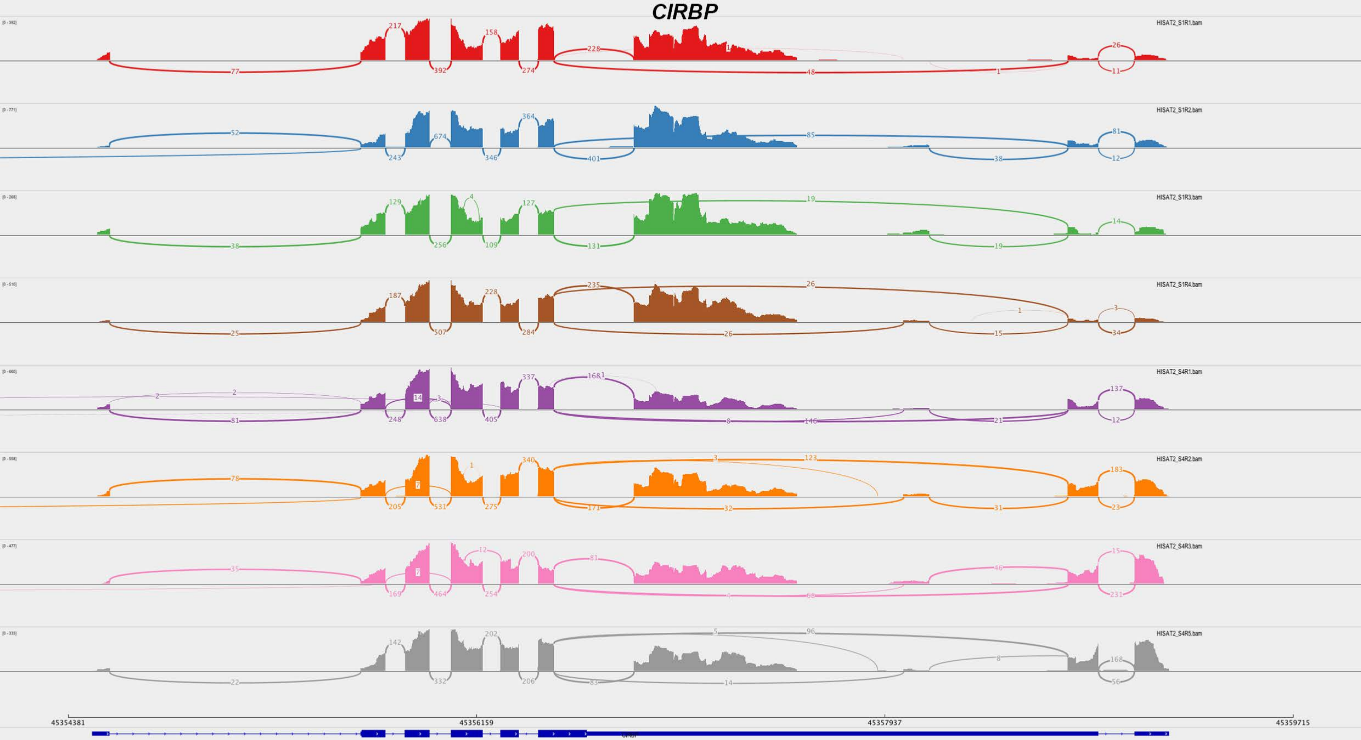

CIRBP
HISAT2_S1R1.bam
HISAT2_S1R2.bam
HISAT2_S1R3.bam
HISAT2_S1R4.bam
HISAT2_S4R1.bam
HISAT2_S4R2.bam
HISAT2_S4R3.bam
HISAT2_S4R5.bam
45354381
45356159
45357937
45359715

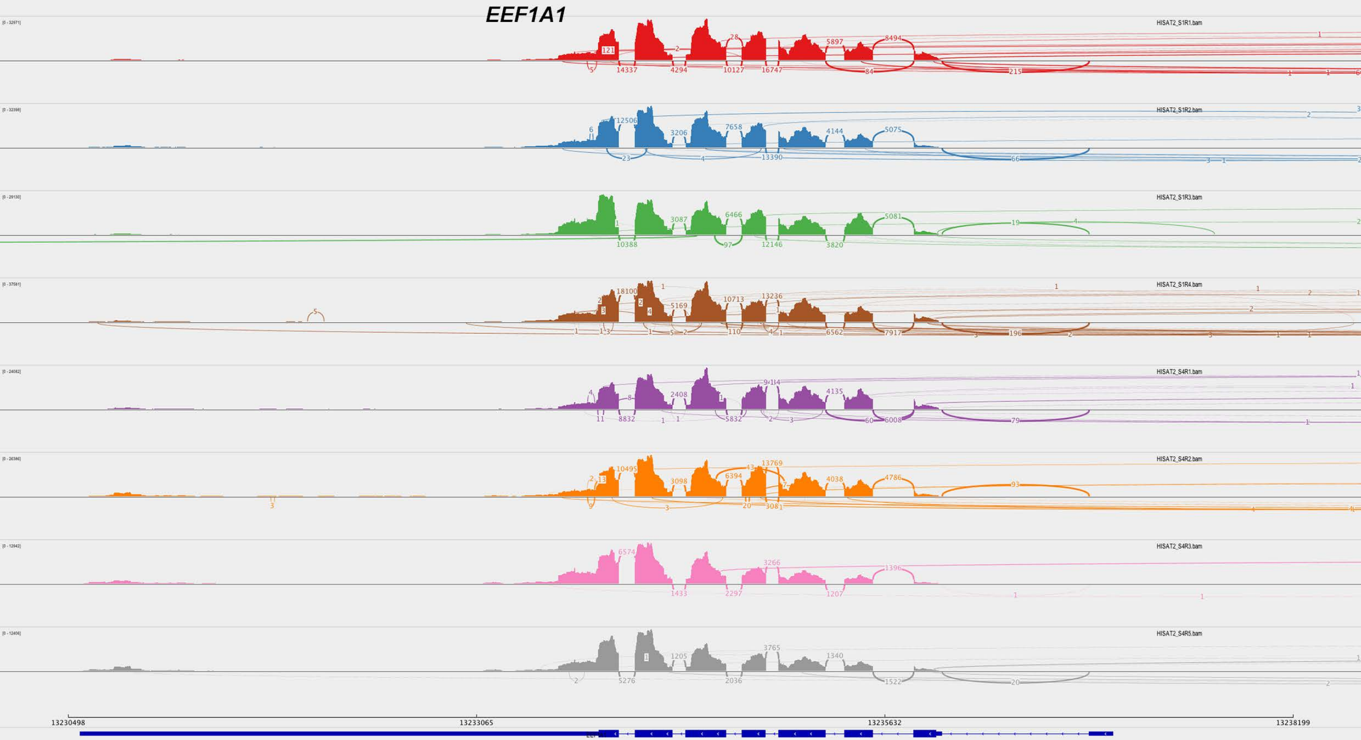

EEF1A1
HISAT2_S1R1.bam
HISAT2_S1R2.bam
HISAT2_S1R3.bam
HISAT2_S1R4.bam
HISAT2_S4R1.bam
HISAT2_S4R2.bam
HISAT2_S4R3.bam
HISAT2_S4R5.bam
13230498
13233065
13235632
13238199

EEF1G

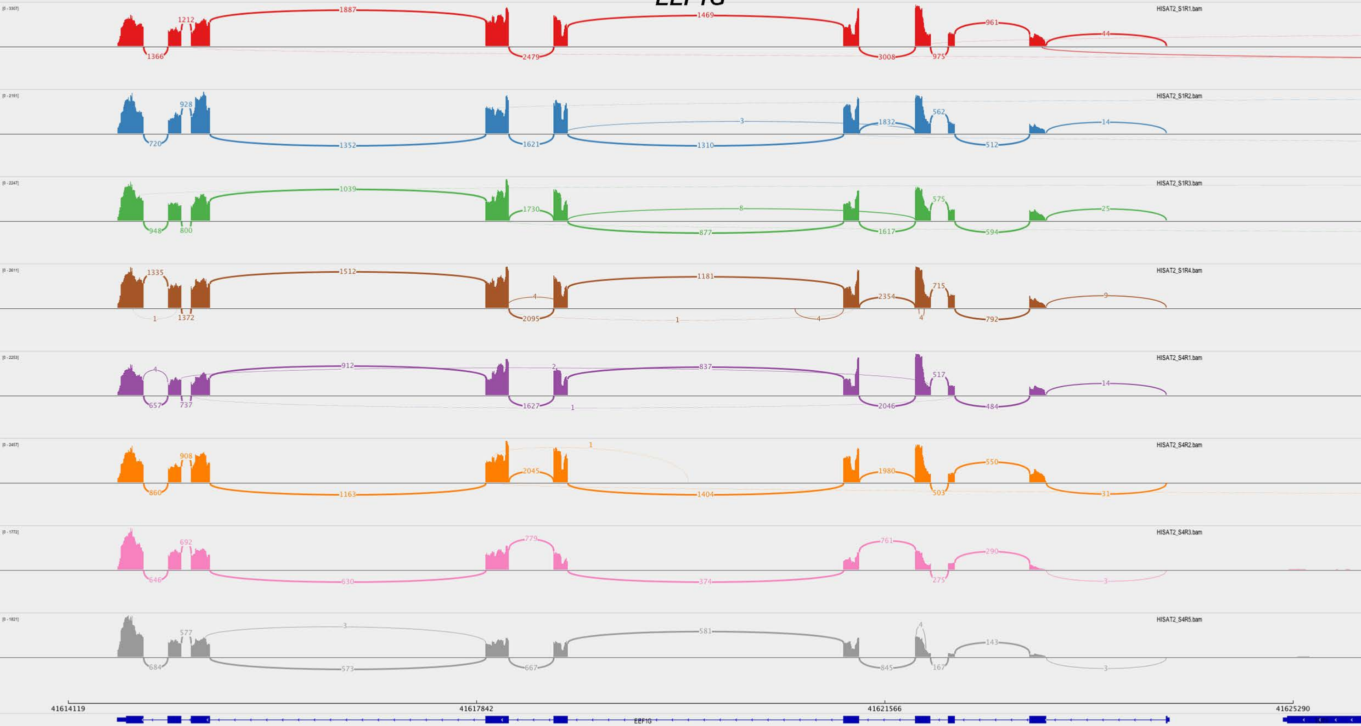
HISAT2_S1R1.bam
HISAT2_S1R2.bam
HISAT2_S1R3.bam
HISAT2_S1R4.bam
HISAT2_S4R1.bam
HISAT2_S4R2.bam
HISAT2_S4R3.bam
HISAT2_S4R5.bam
41614119
41617842
41621566
41625290
EEF1G

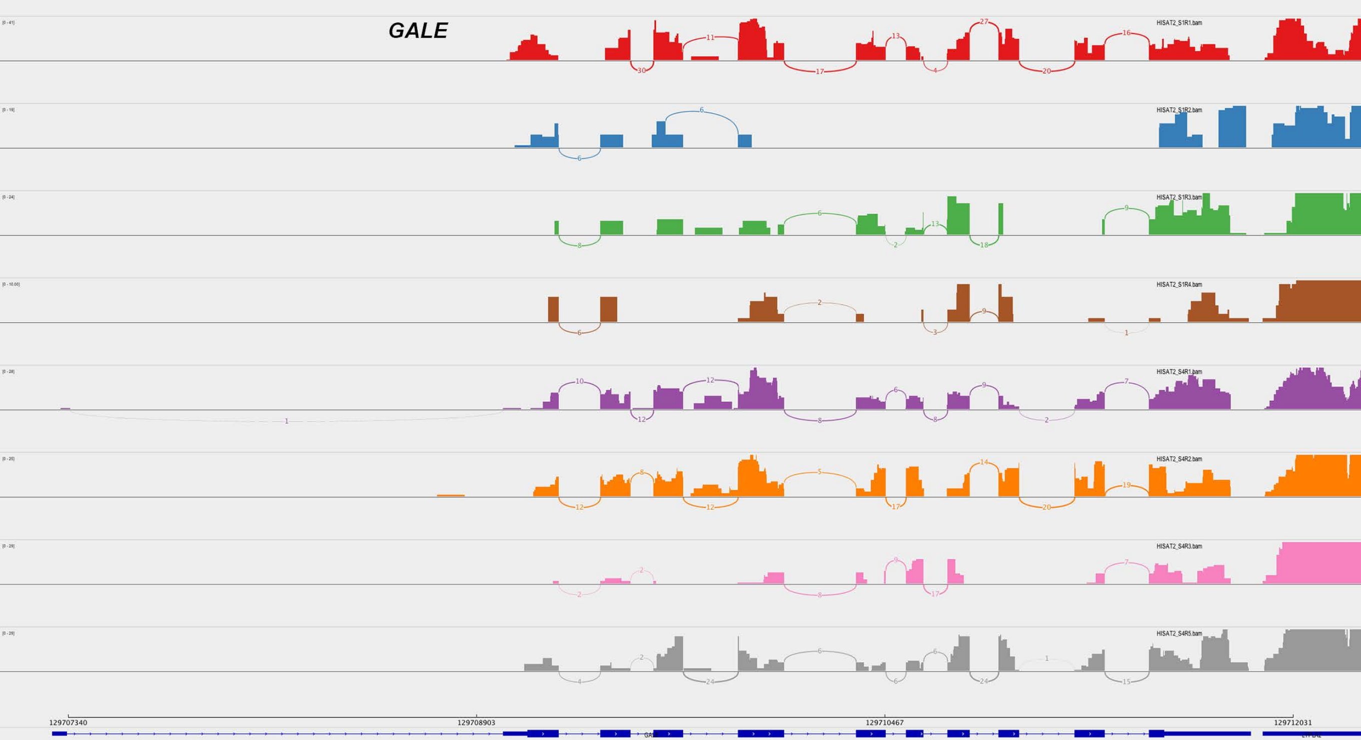
GALE
[0 - 41]
HISAT2_S1R1.bam
[0 - 19]
HISAT2_S1R2.bam
[0 - 24]
HISAT2_S1R3.bam
[0 - 10.00]
HISAT2_S1R4.bam
[0 - 28]
HISAT2_S4R1.bam
[0 - 25]
HISAT2_S4R2.bam
[0 - 29]
HISAT2_S4R3.bam
[0 - 29]
HISAT2_S4R5.bam
129707340
129708903
129710467
129712031

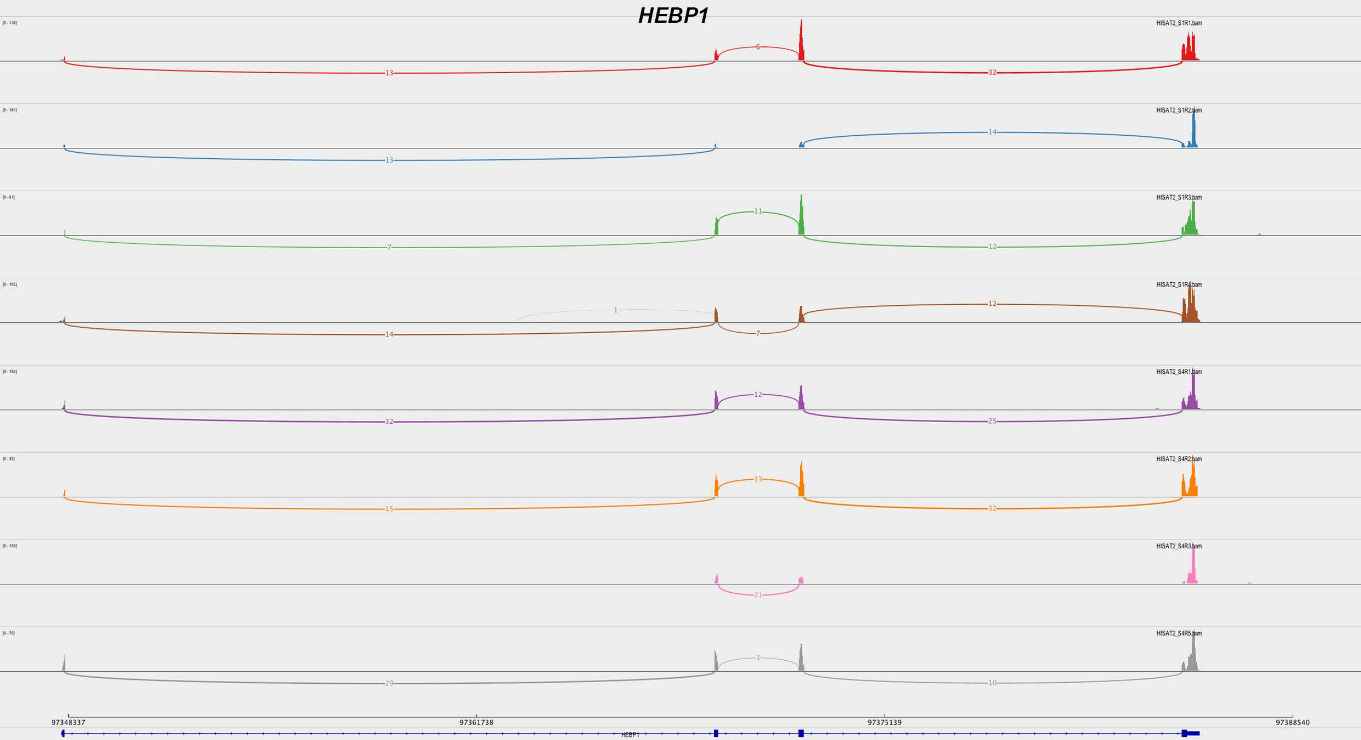
HEBP1
HISAT2_S1R1.bam
6
13
32
HISAT2_S1R2.bam
14
13
HISAT2_S1R3.bam
11
7
12
HISAT2_S1R4.bam
1
12
14
7
HISAT2_S4R1.bam
12
32
25
HISAT2_S4R2.bam
13
15
32
HISAT2_S4R3.bam
21
HISAT2_S4R5.bam
3
29
10
97348337
97361738
97375139
97388540
HEBP1

HSP90AA1

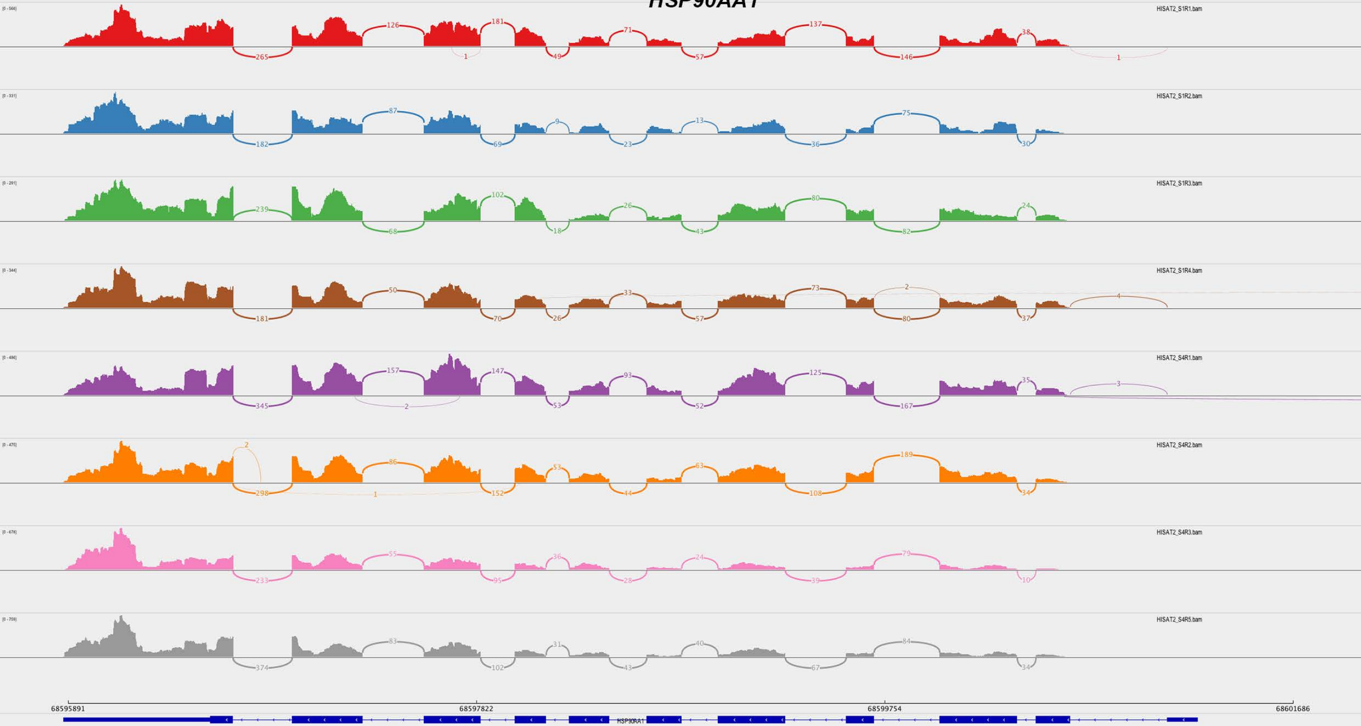
HISAT2_S1R1.bam
HISAT2_S1R2.bam
HISAT2_S1R3.bam
HISAT2_S1R4.bam
HISAT2_S4R1.bam
HISAT2_S4R2.bam
HISAT2_S4R3.bam
HISAT2_S4R5.bam
68595891
68597822
68599754
68601686
HSP90AA1

OVGP1

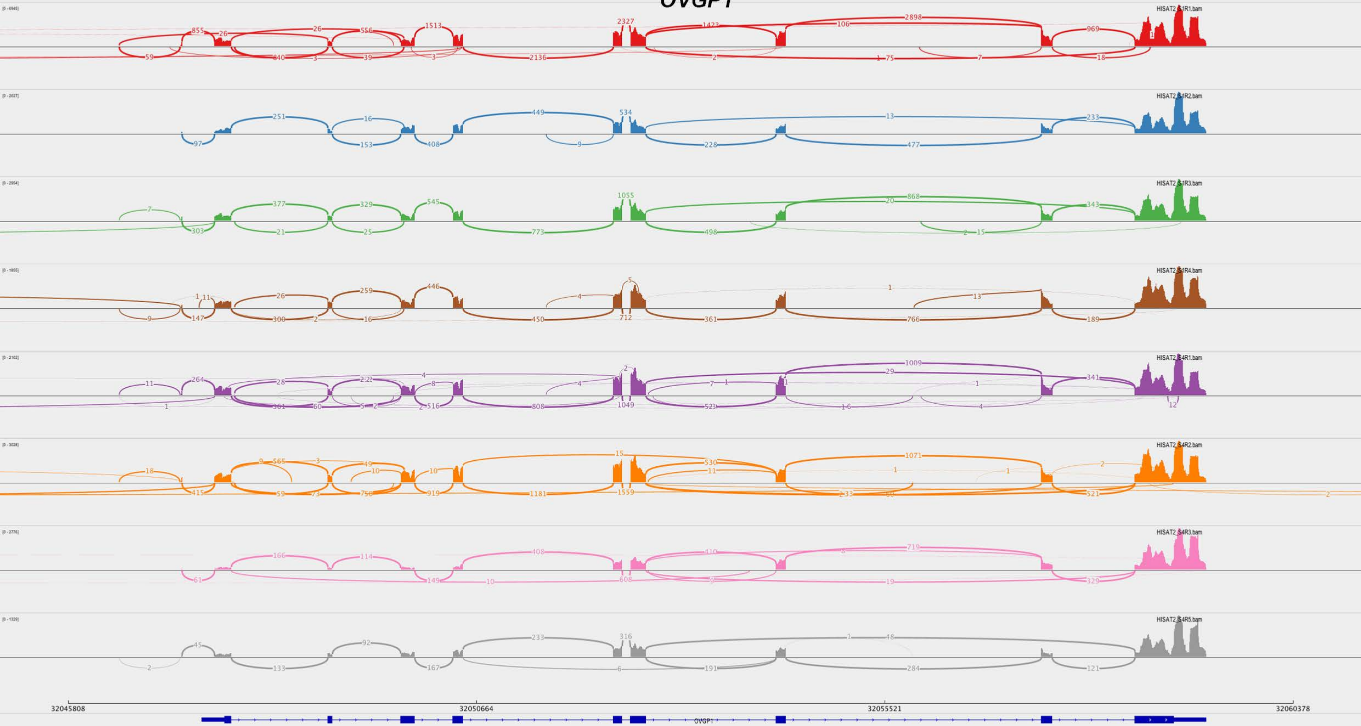

HISAT2_S1R1.bam
HISAT2_S1R2.bam
HISAT2_S1R3.bam
HISAT2_S1R4.bam
HISAT2_S4R1.bam
HISAT2_S4R2.bam
HISAT2_S4R3.bam
HISAT2_S4R5.bam
32045808
32050664
32055521
32060378
OVGP1

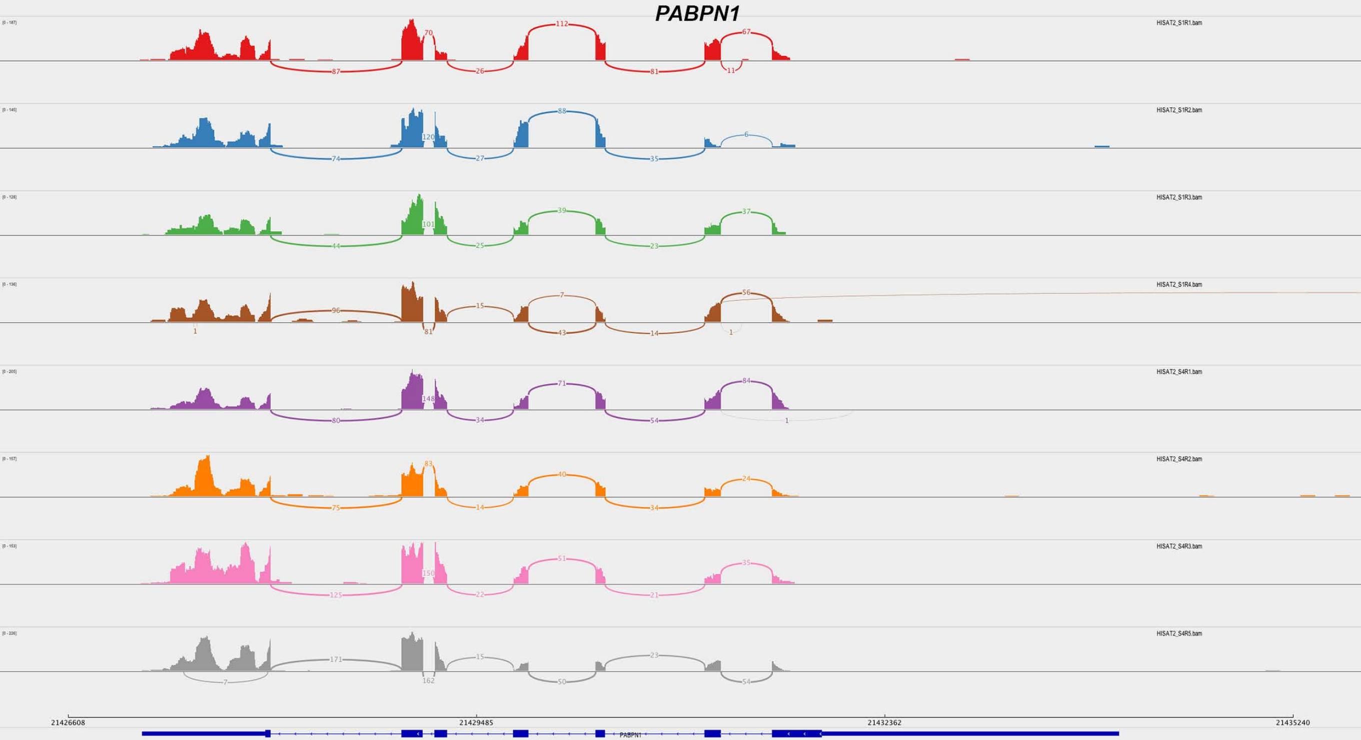
PABPN1
HISAT2_S1R1.bam
HISAT2_S1R2.bam
HISAT2_S1R3.bam
HISAT2_S1R4.bam
HISAT2_S4R1.bam
HISAT2_S4R2.bam
HISAT2_S4R3.bam
HISAT2_S4R5.bam
21426608
21429485
21432362
21435240
PABPN1

RACK1

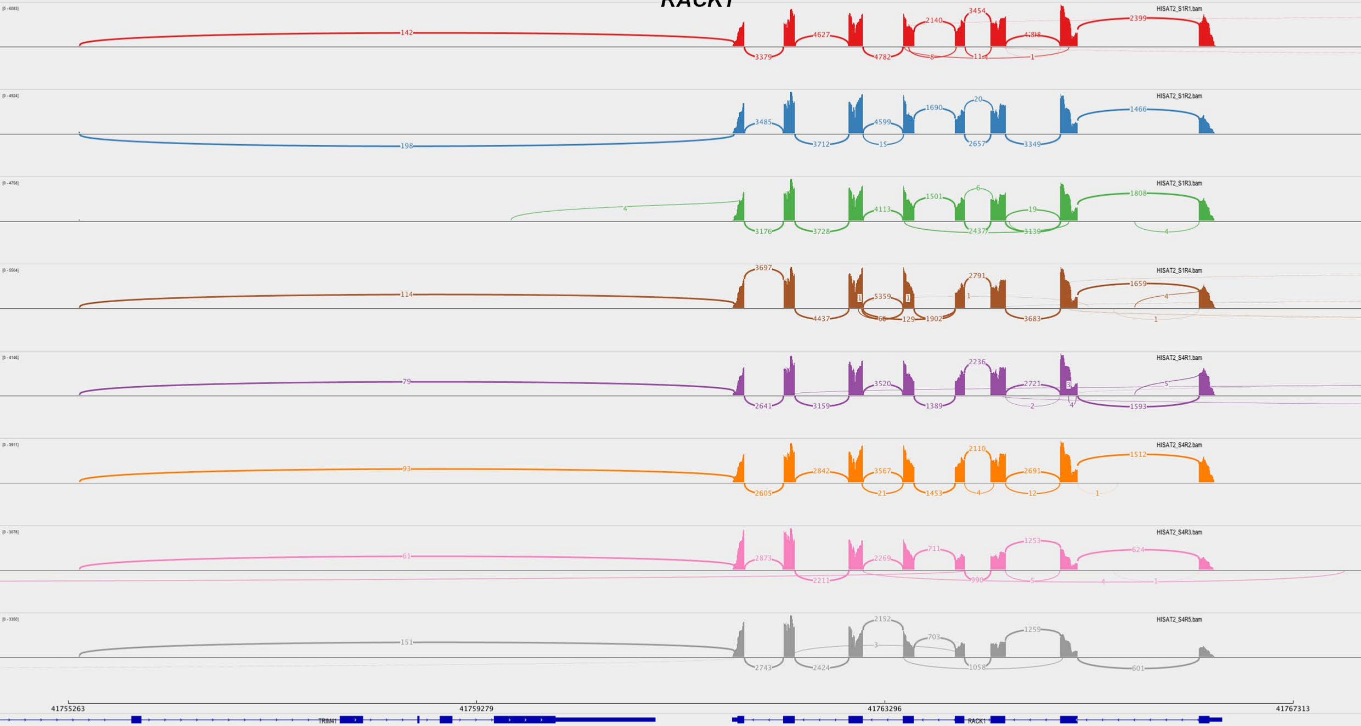

HISAT2_S1R1.bam
142
3379
4627
4782
2140
3454
2399
HISAT2_S1R2.bam
198
3485
3712
4599
15
1690
20
2657
3349
1466
HISAT2_S1R3.bam
4
3176
3728
4113
1501
6
2437
19
3139
1808
HISAT2_S1R4.bam
114
3697
4437
5359
129
1902
2791
3683
1659
HISAT2_S4R1.bam
79
2641
3159
3520
1389
2236
2721
1593
HISAT2_S4R2.bam
93
2605
2842
3567
21
1453
2110
2691
12
1512
HISAT2_S4R3.bam
61
2873
2211
2269
711
990
1253
624
HISAT2_S4R5.bam
151
2743
2424
2152
703
1058
1259
601
41755263
41759279
41763296
41767313
TRIM41
RACK1

RPL6

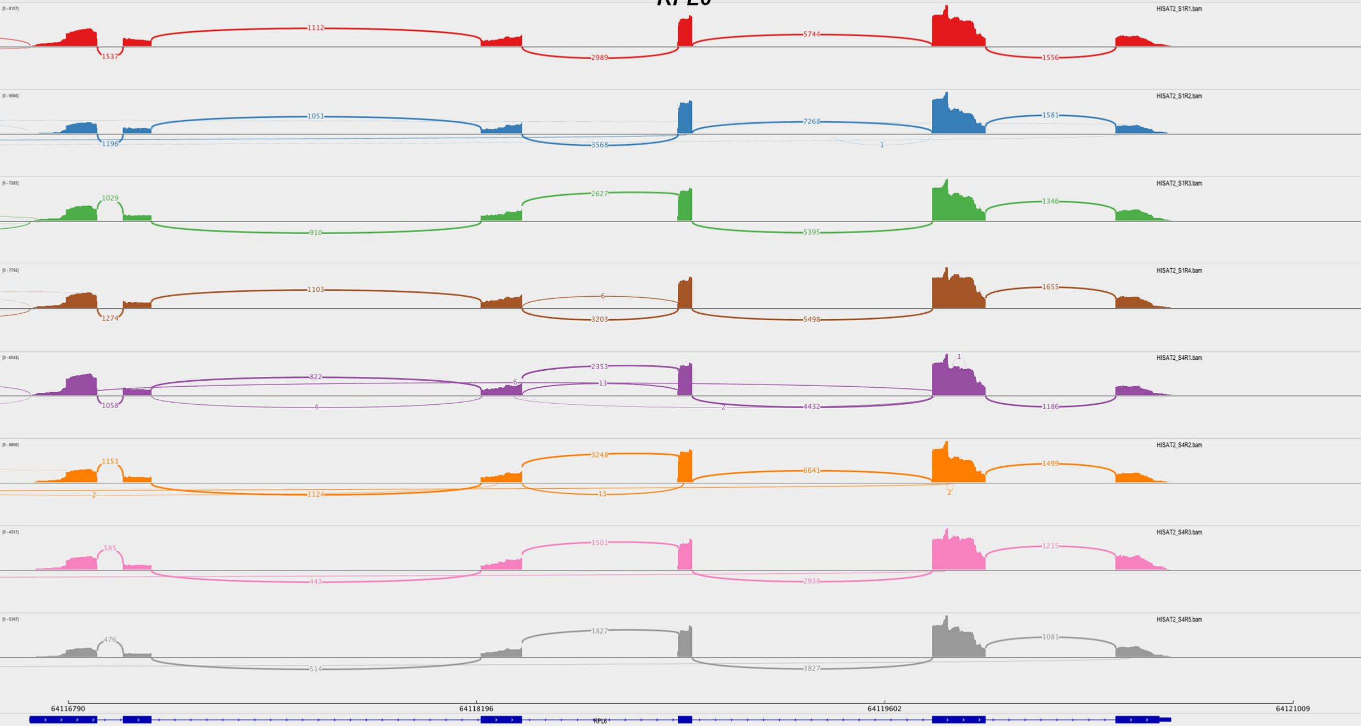

HISAT2_S1R1.bam
1112
5744
1537
2989
1556
HISAT2_S1R2.bam
1051
7268
1581
1196
3568
1
HISAT2_S1R3.bam
1029
2627
1346
910
5395
HISAT2_S1R4.bam
1103
6
1655
1274
3203
5498
HISAT2_S4R1.bam
822
2353
1
6
13
1058
4
2
4432
1186
HISAT2_S4R2.bam
1153
3248
6641
1499
2
1124
13
2
HISAT2_S4R3.bam
585
1501
1215
443
2938
HISAT2_S4R5.bam
476
1827
1081
514
3827
64116790
64118196
64119602
64121009
RPL6

RPS8

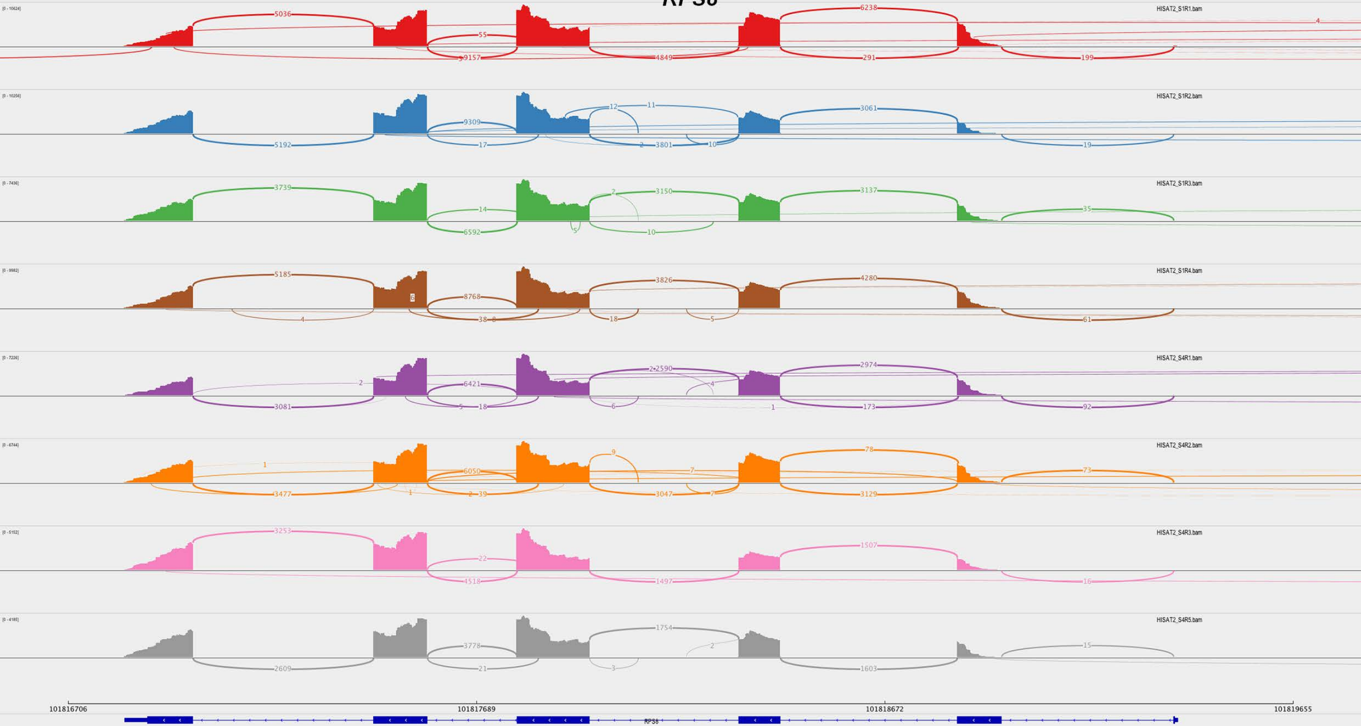
HISAT2_S1R1.bam
HISAT2_S1R2.bam
HISAT2_S1R3.bam
HISAT2_S1R4.bam
HISAT2_S4R1.bam
HISAT2_S4R2.bam
HISAT2_S4R3.bam
HISAT2_S4R5.bam
101816706
101817689
101818672
101819655
RPS8
